# An inactivated vaccine against acquired *Toxoplasma gondii* infection in pigs as a tool to minimize the zoonotic transmission risk

**DOI:** 10.1186/s13567-025-01645-2

**Published:** 2025-10-30

**Authors:** Andrea Largo-de la Torre, Roberto Sánchez-Sánchez, Carlos Diezma-Díaz, Ignacio Ferre, Javier Regidor-Cerrillo, Luis Miguel Ortega-Mora

**Affiliations:** 1 SALUVET-Innova, Faculty of Veterinary Sciences, Complutense University of Madrid, Ciudad Universitaria S/N, 28040 Madrid, Spain; 2 SALUVET, Animal Health Department, Faculty of Veterinary Sciences, Complutense University of Madrid, Ciudad Universitaria S/N, 28040 Madrid, Spain

**Keywords:** Swine, multi-stage vaccine, infection control food safety, zoonoses, One Health approach

## Abstract

*Toxoplasma gondii*, a zoonotic protozoan parasite, represents a major threat to public health and food safety worldwide, particularly through the consumption of undercooked or raw pork containing tissue cysts. Controlling *T. gondii* infection in pigs is therefore critical to reduce zoonotic transmission. This study evaluated a novel inactivated vaccine formulated with antigens from the Type III TgPigSp1 isolate, comprising both tachyzoite and bradyzoite stages, and adjuvanted with Quil-A. Safety, immunogenicity, and efficacy were assessed first in a murine model as proof of concept and subsequently in piglets challenged with 1000 oocysts of the heterologous Type II-PRU TgShSp1 isolate. The vaccine showed no systemic adverse effects in either model, and an absence of local reactions was observed in piglets. In mice, vaccination elicited strong parasite-specific IgG responses and significantly reduced the parasite burden in brain and muscle tissues by more than 80%. In piglets, robust humoral and cellular immune responses were induced, as evidenced by elevated IgG and IFN-γ levels after the booster vaccination. Following challenge, the parasite load was reduced by ≥ 95% in target muscles, and viable *T. gondii* cysts were undetectable in at least 50% of vaccinated animals, as confirmed by bioassay. Molecular detection methods further supported the significant reduction in the parasite DNA content. These results indicate that this inactivated multistage vaccine represents a safe, immunogenic, and effective strategy to reduce tissue cyst formation and offer a promising tool within a One Health approach to improve food safety and reduce the burden of toxoplasmosis in both animal and human populations.

**Supplementary Information:**

The online version contains supplementary material available at 10.1186/s13567-025-01645-2.

## Introduction

The protist parasite *Toxoplasma gondii*, which is capable of infecting most warm-blooded animals, including humans and livestock, poses significant challenges to public health and animal production. This obligate intracellular parasite has a complex life cycle comprising three infectious stages for intermediate hosts: sporozoites within environmentally resistant oocysts, rapidly proliferating tachyzoites, and bradyzoites encysted in tissues. While oocysts are exclusively produced by definitive hosts (members of the Felidae family, such as domestic cats), intermediate hosts, including humans and food-producing animals such as pigs, sheep, and goats, often remain chronically infected, harboring cysts in immune-privileged tissues such as the brain, heart, and skeletal muscles, including humans [1].

Although *Toxoplasma gondii* infection is usually asymptomatic in immunocompetent individuals, reactivation in immunocompromised hosts can lead to severe, potentially fatal conditions such as encephalitis and retinitis [2, 3]. Chronic infection has also been linked to neuropsychiatric disorders in healthy individuals [2, 3]. Ocular toxoplasmosis may arise from congenital or acquired infection and can cause progressive vision loss [4]. In pregnant women, the primary infection may result in vertical transmission with serious fetal or neonatal outcomes [5]. As a result, toxoplasmosis represents a major global health concern, affecting more than a third of the global population and ranking among the top foodborne diseases in Europe [5].

Pigs play a critical role in the transmission of *T. gondii* to humans, as pork is one of the most widely consumed meats globally. The parasite forms tissue cysts in pig muscles, which can remain infectious for extended periods. The consumption of undercooked or raw pork containing these cysts is a major route of human infection [6, 7]. Different studies suggest that pork consumption may account for between 30 and 63% of human toxoplasmosis cases in certain European regions [3, 8–10]. In pigs, *T. gondii* infection is often subclinical [11]. A global meta-analysis estimated a pooled seroprevalence of *T. gondii* in pigs of approximately 19%, with significant regional differences linked to factors such as latitude and temperature [12, 13]. However, comparisons are limited by the use of nonstandardized methods and the lack of data from intensive farms. These findings highlight the importance of controlling *T. gondii* in pigs to protect public health and reduce economic losses.

Several vaccine strategies have been tested in pigs against *T. gondii* infection to reduce tissue cyst formation and diminish the risk of transmission through pork consumption. Live attenuated vaccines, such as the “incomplete” S48 strain, have shown the best efficacy in reducing the cyst burden under controlled conditions [14] but raise safety concerns due to the potential risk of reversion to a virulent state and accidental infection of operators, in addition to limited shelf life and market distribution, which limit their implementation in pig production. Recombinant protein-based, DNA-vectored and inactivated vaccines offer safer alternatives; however, their efficacy has been very inconsistent [15–17]. Therefore, a safe, effective, and stable vaccine capable of inducing long-lasting immunity, significantly reducing the tissue cyst burden, and is suitable for large-scale implementation in pig production systems is critically needed to minimize the zoonotic risk associated with pork consumption.

The present study explores a new multistage inactivated vaccine containing both tachyzoite and bradyzoite antigens of *T. gondii* against acquired toxoplasmosis in pigs that is designed to ensure safety, scalable production and distribution, which are limited with live vaccines. The vaccine antigen is obtained from *T. gondii* capable of spontaneously forming mature tissue cysts in Vero-81 cell cultures suitable for vaccine production [18] and is then inactivated, concentrated and solubilized physicochemically prior to adjuvantation with *Quillaja saponaria* saponin (Quil-A®) for cellular and humoral immune stimulation. The vaccine formulation was evaluated against acquired *T. gondii* infection in mice as proof of concept and, finally, in a well-characterized pig model of *T. gondii* infection. Ultimately, the goal of this study was to provide a safe, effective, and marketable vaccination strategy for pigs that contributes to the control of *T. gondii* infection in livestock and reduces the zoonotic risk associated with pork consumption.

## Materials and methods

### Ethics statements

The animal procedures were approved by the Animal Welfare Committee of the Community of Madrid, Spain (PROEX 062/19, PROEX 290.4/20, and PROEX 293.7/20) and were conducted in compliance with Spanish and EU regulations (Law 3/2007, R.D. 53/2013, and Council Directive 2010/63/EU).

### Parasites

Antigenic materials for vaccine production were obtained from Type II-PRU TgShSp3 (ToxoDB#3) and Type III TgPigSp1 (ToxoDB#2) *T. gondii* isolates. The isolates were used between passages 29 and 33 after isolation [19, 20] and were maintained in vitro in Vero-81 cell cultures using DMEM (D5796, Sigma-Aldrich, Madrid, Spain) supplemented with 1% fetal bovine serum (P30-3306, Lonza, Basel, Switzerland) at 37 °C with 5% CO_2_ [21]. The parasites were recovered from flasks immediately after cell infiltration, purified and aliquots of 10^8^ parasites were stored at −80 °C until vaccine antigen preparation, as previously described [21].

Sporulated oocysts from the Type II TgShSp1 isolate (ToxoDB#3) were used for the experimental challenge in mouse and pig vaccine trials. Oocysts were produced in a kitten and sporulated by resuspension in 2% H_2_SO_4_ for 4 days at room temperature, as previously described [22]. The desired concentrations of sporulated oocysts were prepared by dilution in PBS and stored at 4 °C for up to 2 years [21]. The infectivity of sporulated oocysts for vaccine trials was confirmed in a mouse model of infection [23].

### Preparation of the vaccine antigen: vaccine antigen inactivation, vaccine antigen characterization and vaccine formulation

#### Vaccine antigen production

Vaccine antigens were obtained from 1 × 10^8^ *T. gondii* parasites of the two isolates at passages 29–33 in Vero cells and maintained at -80 °C. Parasites were resuspended in 600 μL of PBS with 0.5% (v/v) protease inhibitor (protease inhibitor cocktail, Sigma-Aldrich), and once the mixture was resuspended, 300 μL of 60% sucrose (w/v in PBS) was added to the suspension to obtain a final concentration of 20% (w/v) sucrose in the mixture. Afterward, the suspension was centrifuged (10 000 × *g*, 60 min, 4 °C), and the pellet was resuspended in 0.1 mL of a 1% Igepal®Ca-630 (Sigma‒Aldrich) solution (v/v in ultrapure water) supplemented with 0.5% of the same protease inhibitor for solubilization of the components. The suspension was maintained under constant agitation on a digital rotator for 18–24 h at 4 °C to ensure complete homogenization. The protein concentration was determined using the Bradford method. Finally, the antigens were aliquoted and stored at −80 °C for characterization studies and vaccine formulation.

#### Vaccine antigen assay for inactivation

Parasite inactivation of the vaccine antigen was confirmed by culturing the treated parasites in Vero cells in vitro. After treatment with sucrose, a portion of the parasites from each production batch was resuspended in PBS instead of the Igepal®Ca-630 solution, and a volume containing 10^7^ parasites was inoculated on a Vero cell monolayer in a T25 flask (MOI of 10:1) and maintained successively in three blind passages every 7 days by inoculating whole-cell cultures in a new T25 flask with a Vero cell monolayer as described above (DMEM supplemented with 1% fetal bovine serum at 37 °C with 5% CO_2_). After three blind successive culture passages, the cell monolayer was recovered with a cell scraper and centrifuged at 1350 × *g* for 10 min, and the pellet was used for DNA extraction and parasite detection by PCR (see parasite quantification).

#### Vaccine antigen characterization

The vaccine antigen composition was determined via a quantitative label-free comparative proteomic analysis after liquid chromatography‒tandem mass spectrometry (LC‒MS/MS). The LC‒MS/MS analysis was performed with 100 µg of protein from four samples of vaccine antigen obtained from the TgPigSp1 and TgShSp3 isolates of batches produced at passages 29–30 (biological replicates). An additional two technical replicates of three TgShSp3 biological replicates were included in the analyses. Briefly, all samples were purified via SDS-PAGE and digested in a trypsin solution. Eluted peptides were analyzed by nano-LC‒MS/MS using a nano-Easy-nLC 1000 coupled to a high-resolution mass spectrometer (Q Exactive HF; Thermo Scientific) and detected with a resolution of 60 000 in full-scan MS mode over an m/z mass range of 300–800 Da. Peptides were identified from MS/MS spectra through Proteome Discoverer 2.4 (Thermo Scientific) using the licensed version of the search engine MASCOT 2.6.1 (Matrix Science, London, UK). Tandem MS/MS data were searched against a database with predicted sequences of *T. gondii* downloaded in August 2021 from TOXODB for the ME49 isolate (*Toxoplasma gondii* 51: ME49; 8322 sequences) [24] for data from the Type II TgShSp3 isolate and for the VEG isolate (*Toxoplasma gondii* 51:VEG; 8410 sequences) [25] for data from the Type III TgPigSp1, together with a database of *Chlorocebus sabaeus* from UniProt (19229 sequences) [26] and the database Contaminants (247 sequences) from the Max Planck Institute of Biochemistry [27]. Proteins that were differentially abundant (fold change ≥ 2 for more abundant and ≤ 0.5 for less abundant; log_2_ ratio ≥ 1 and log_2_ ratio ≤ −1, respectively, and with a coefficient of variation < 30) between the TgPigSp1 and TgShSp3 vaccine antigens were also identified.

The immunoreactive profiles of the vaccine antigens of the TgPigSp1 or TgShSp3 isolates were also studied by immunoblotting to evaluate variations among batches of production, isolates and the presence of the specific bradyzoite protein TgBAG1. Briefly, immunoreactive profiles were studied in membranes after proteins in 10 µg of vaccine antigens from different batches of production were separated on 12% SDS-PAGE gels using a Mini-PROTEAN Tetra Cell system (Bio-Rad), transferred to membranes, and detected with a hyperimmune mouse antiserum directed against *T. gondii* (1:50) or monoclonal mouse serum against TgBAG1 (1:100) (GenScript, New Jersey, USA). Antigen profiles were analyzed by comparing the relative molecular weights of the samples with Quantity One software (Bio-Rad).

The materials and methods used for this experiment are broadly described in the Supporting information (Additional file 6).

#### Vaccine formulation

For vaccine trials in mice and piglets, the vaccine was prepared immediately before inoculation, combining the vaccine antigens from the TgPigSp1 and TgShSp3 isolates with the required amount of adjuvant.

Antigen doses containing 5 to 40 µg of protein, as determined by the Bradford assay, were prepared from a mixture of vaccine antigens from passages 29 to 30. These were combined with 100 µg (for mice) or 300 µg (for piglets) of Quil-A® saponin (InvivoGen, Toulouse, France), and the final volume was adjusted with PBS (pH 7.2–7.4) to 0.2 mL for the mice and 1 mL for the piglets.

### Evaluation strategy and selection criteria for vaccine safety, immunogenicity and efficacy

Prior to testing the vaccine prototype, a sequential evaluation strategy was established to progressively assess its safety, immunogenicity, and efficacy. The initial evaluation was conducted using a standardized murine model of acquired toxoplasmosis, which served as proof of concept. This model allows for controlled experimental conditions, facilitating reproducibility and enabling comparisons of immune responses and levels of protection across different vaccine formulations. Once preliminary evidence of safety, immunogenicity, and efficacy was obtained in mice, the prototype was subsequently evaluated in the natural host species, pigs, which is essential for assessing vaccine performance under more biologically and epidemiologically relevant conditions.

The safety criteria included the absence of severe systemic adverse effects and the absence of intense or extensive local reactions at the injection site. Mild to moderate local inflammation or lesions, as well as low-grade hyperthermia, were anticipated and considered acceptable. In terms of immunogenicity, the vaccine was expected to elicit both humoral and cellular immune responses in a balanced manner, avoiding excessive polarization toward either response. Subsequently, efficacy was evaluated by determining parasite viability and burden in target tissues, as well as clinical parameters, following exposure. An expected reduction of at least 80% in parasite detection/load in vaccinated animals was established as a predefined efficacy goal. These criteria guided the experimental design and interpretation of the results in both the murine and porcine models.

### Proof of concept: evaluation of the safety, immunogenicity and efficacy of the vaccine prototype in a murine model

A standardized acquired infection mouse model using sporulated oocysts of the Type II-PRU TgShSp1 isolate was used [22, 23].

#### Animals

Seventy-four 6-week-old female Swiss mice purchased from Janvier Labs (Le Genest-Saint-Isle, France) were used for the vaccine trial. The results of a routine screen conducted by the supplier confirmed that the animals were free of common viral, parasitic, and bacterial pathogens. The animals were maintained in a regulated environment with equal periods of light and darkness and had unrestricted access to food and water.

#### Experimental design, clinical monitoring and sample collection

**Figure 1:**
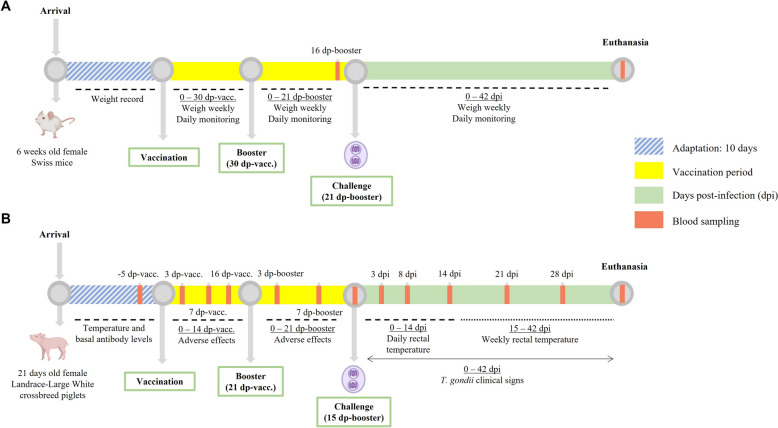
**Experimental designs for the vaccine trial against *T. gondii* in mice (A) and piglets (B).** The figure outlines the timeline, including the immunization schedule, challenge with *T. gondii* and all procedures performed during the experiment. Dp-vacc.: days post-vaccination, dp-booster: days post-booster, dpi: days post-infection.

The experimental design is represented in Figure 1A. The variables studied were antigenic variations (TgShSp3 vs. TgPigSp1) and antigen dose (20 µg vs. 5 µg). The mice were divided into eight groups: Group 1 (G1–20 mix) received a vaccine containing 20 µg of a 50:50 mixture of TgShSp3 and TgPigSp1 antigens per dose; Group 2 (G2–20 Sp3) received 20 µg of TgShSp3 antigen per dose; Group 3 (G3–5 Sp3) received 5 µg of TgShSp3 antigen; Group 4 (G4–20 Pig1) received 20 µg of TgPigSp1 antigen; Group 5 (G5–5 Pig1) received 5 µg of TgPigSp1 antigen; and Group 6 (G6–Quil-A®) received adjuvant only (100 µg Quil-A®). Groups G1 to G6 consisted of ten mice each (*n* = 10). Group 7 (G7–PBS, *n* = 9) and the sentinel Group 8 (G8–Sentinel, *n* = 5) received the same volume of PBS. The mice were vaccinated via the subcutaneous route.

The mice were vaccinated on day 0 and received a booster at 30 days post-vaccination. Side effects and local reactions were monitored 14 days after vaccination and booster immunization using the scores shown in Additional file 3. All groups, except G8—Sentinel, were orally challenged with 100 sporulated oocysts of the TgShSp1 isolate at 21 days after the booster immunization. After challenge, the mice were monitored daily for 42 days for clinical signs compatible with toxoplasmosis and scored as previously reported [23] and body weight was recorded weekly to assess safety. Mice exhibiting severe signs (score = 4) were humanely euthanized by gradual exposure to CO_2_, in accordance with the ethical guidelines for animal welfare.

Blood samples were collected via puncture in the submandibular venous sinus 5 days prior to the challenge to evaluate the immune response after vaccination and the booster immunization. On day 42 post-challenge, the mice were euthanized, and blood was collected via direct cardiac puncture for assessment of the *T. gondii*-specific humoral response by detecting IgG, IgG1 and IgG2a antibody levels. Tissues such as the brain, tongue, quadriceps muscle, and *Longissimus dorsi* muscle were collected and stored at −80 °C for *T. gondii* genomic DNA quantification by PCR. In addition, the number of cysts in the brain was determine by microscopy and counting.

#### Evaluation of the humoral immune response in mice

*T. gondii*-specific IgG, IgG1 and IgG2a levels were measured, as previously described [23]. The samples were considered positive (i.e., the mice had seroconverted) if the relative index percentage (RIPC) was > 28.67.

#### Detection and quantification of T. gondii DNA in mouse tissues

Genomic DNA was extracted from 50–100 mg of each mouse tissue sample using a commercial Maxwell RSC Tissue DNA Kit (Promega, Wisconsin, USA) according to the manufacturer's instructions. *T. gondii* DNA detection and quantification were performed as described previously [21]. One hundred micrograms of DNA were used for the quantification of parasite DNA in the sample, and the standard curve was constructed as reported in [21] and diluted in PCR-negative mouse DNA. The slope (S between −3.57 and −3.18) and regression coefficient (R^2^ > 0.993) of the standard curves were also calculated to estimate the efficiency of the assay.

#### Analysis of the cyst burden in the brains of the mice

Half of the fresh brain from each mouse was weighed and set aside for counting the cysts [23]. Tissue cyst numbers were determined using DBL-FICT staining to enhance cyst visualization and facilitate counting under the microscope, particularly in samples with an expected low parasite burden. Each half of the brain was homogenized in 500 µL of PBS through successive passes with needles of different diameters (18G–25G) and centrifuged at 500 × *g* for 10 min. The sediment was washed three times in PBS by centrifugation at 500 × *g* for 10 min and fixed with 4.5 mL of methanol at −20 °C for 20 min. After being washed again three times with PBS, the samples were labeled with fluorescein isothiocyanate (FITC)-conjugated *Dolichos biflorus* lectin (DBL-FITC, Vector Laboratories) at a dilution of 1:50 for 1 h with shaking. The cysts were recovered by centrifugation as described above and washed again three times with PBS, after which they were suspended in a final volume of 500 µL of PBS. The number of cysts was determined in at least three aliquots of 25 µL of the brain homogenate from each mouse by counting labeled structures compatible with cysts under a fluorescence microscope at 40x. The total number of cysts per mg of tissue was calculated according to the final volume and tissue weight (mg) of the brain sample examined.

### Pig trial: evaluation of safety, immunogenicity and efficacy in a piglet model

A standardized piglet model of acquired infection using *T. gondii* sporulated oocysts of the Type II-PRU TgShSp1 isolate was used [21].

#### Animals

Twenty-seven weaned Landrace–Large White crossbred female piglets, aged 21 days, were used for the vaccine trial. Their procurement and health status were assessed as described in [21].

#### Experimental design, clinical monitoring and sample collection

The experimental design is illustrated in Figure 1B. After the adaptation period, the piglets were assigned into four groups: Group 1 (G1–20, *n* = 8), which received a 20 µg dose of the vaccine; Group 2 (G2–40, *n* = 8), which received a 40 µg dose of the vaccine (both derived from the TgPigSp1 vaccine antigen); Group 3 (G3–PBS, *n* = 8); and Group 4 (G4–Sentinel, *n* = 3), which received PBS. Following a 7-day adaptation period, 28-day-old piglets were intramuscularly vaccinated and received a booster 21 days later. Each group of animals was housed separately in environmentally controlled boxes, provided with bedding, and given unrestricted access to food and water throughout the experiment.

Safety was assessed by monitoring adverse reactions, such as localized inflammation, redness, or pain at the injection site, as well as systemic reactions or changes in behavior (Additional file 7). The rectal temperature was recorded 2 h after vaccination and daily for 15 days post-vaccination to monitor hyperthermia. A temperature above 40 °C was considered indicative of hyperthermia [28]. Blood samples were collected on days −5, 3, 7, 16, 24, 28, and 36 after vaccination to evaluate the immune response, as previously described [21].

Fourteen days after booster immunization, piglets from the G1–20, G2–40, and G3–PBS groups were orally challenged with 1000 sporulated oocysts of the TgShSp1 isolate (Type II-PRU) suspended in 1 mL of PBS. The inoculum was administered directly into the oral cavity using a syringe, following a standardized model [21]. Piglets voluntarily ingested the entire dose concentrated in a small volume, minimizing animal handling and stress and assuring accurate delivery. Group 4 (G4–Sentinel) received PBS without challenge and served as the unchallenged control group. Appropriate precautions were implemented to prevent cross-contamination of oocysts between boxes. Clinical signs, including diarrhea, anorexia, apathy and behavioral changes, were monitored daily throughout the entire experimental period. The rectal temperature was recorded daily after challenge up to 14 days post-infection and then weekly until the end of the experiment. Blood samples were collected as described above on days 3, 8, 14, 21, 28 and 36 after challenge.

At the end of the experiment (42 days after challenge), the animals were sedated and euthanized according to the procedure in [21]. During necropsy, tissue samples from the brain, heart, and skeletal muscles (including *Longissimus dorsi* and semimembranosus) were collected and stored for parasite detection and quantification, as described in [21].

#### Mouse bioassay

Tissue homogenates were obtained via acid‒pepsin artificial digestion, following the method in [29]. Brain homogenates were prepared similarly, without acid–pepsin treatment [21, 30]. The homogenates were used to inoculate mice and the bioassay was conducted as described in [21]. During necropsy, blood and brain samples from each mouse were collected for IgG and *T. gondii* detection by qPCR, respectively, as described above (see “Evaluation of the humoral immune response in mice” and “Detection and quantification of T. gondii DNA in mouse tissues” sections). Mice that tested positive by qPCR and/or serological tests were considered infected. Additionally, in animals that tested positive by qPCR but remained IgG negative, exposure to *T. gondii* was later confirmed by evaluating IgM responses under the ELISA conditions described above for IgG determination but using a goat anti-mouse IgM antibody (1:500; AP128P, Sigma Aldrich).

#### Humoral and cellular immune responses in piglets

*T. gondii*-specific humoral responses were evaluated in piglets using an *in-house* ELISA to determine IgG, IgG1 and IgG2 levels, as previously described [21].

*T. gondii* cellular immune responses were assessed by measuring antigen-specific IFN-γ production in the supernatants of stimulated blood cells in vitro using a commercial porcine enzyme immunoassay kit, following the detailed protocol described in [21]. Whole blood was used instead of isolated peripheral blood mononuclear cells (PBMCs) to preserve the physiological cellular environment and enable simultaneous assessment of responses involving all immune cell populations, including monocytes and lymphocytes. Appropriate negative controls were included to account for nonspecific responses.

#### T. gondii detection and quantification in piglet tissues

Genomic DNA was extracted from tissue extracts following the method reported in [21]. *T. gondii* DNA in target tissues from piglets was detected using an ITS1 PCR adapted to a single tube format based on previously described procedures [31], and parasite DNA was quantified as previously described [21].

### Statistical analysis

Differences in total protein abundance between the TgPigSp1 and TgShSp3 vaccine antigens were analyzed via a nonparametric Mann‒Whitney test. Individual proteins that were differentially abundant between the TgPigSp1 and TgShSp3 vaccine antigens were analyzed as described above (Additional file 6).

In the mouse trial, mortality rates were analyzed using the Mantel‒Cox log-rank test to compare survival curves. Variations in morbidity scores, parasite burdens in tissues, and cyst numbers in the brain between different groups were analyzed using the nonparametric Kruskal‒Wallis test, followed by Dunn’s test for pairwise comparisons or the Mann‒Whitney test. Antibody levels were analyzed using one-way ANOVA followed by Tukey's multiple comparisons test, and body weight variations between groups were analyzed using two-way ANOVA with repeated measures followed by Tukey’s post hoc test. Spearman’s correlation analyses were performed between the parameters assessed by qPCR and brain cyst counts.

In the piglet trial, rectal temperatures; IgG, IgG1 and IgG2a responses; and IFN-γ levels were analyzed using two-way repeated-measures ANOVA followed by Tukey’s post hoc test. Fisher’s exact test was applied to analyze the frequencies of parasite detection in tissues in the mouse bioassay and PCR analysis. Mortality rates in the mouse bioassay were analyzed using the Mantel–Cox log-rank test to compare the resulting survival curves. The parasite burden in tissues from piglets was analyzed using the nonparametric Kruskal–Wallis test followed by Dunn’s test for comparisons between groups or the Mann–Whitney test.

Statistically significant differences were considered at *p* < 0.05. All statistical analyses were performed and graphical illustrations were created using GraphPad Prism 6 v.6.01 software (San Diego, CA, USA).

## Results

### Vaccine antigen production triggered parasite inactivation for safety, and proteomic characterization revealed a multistage composition with tachyzoite and bradyzoite proteins

The *T. gondii* isolates TgShSp3 (Type II-PRU) and TgPigSp1 (Type III) were selected as sources for vaccine antigen production because of their ability to spontaneously produce mature cysts in vitro. Parasites harvested from host cell cultures were subjected to hyperosmotic shock, followed by treatment with the detergent Igepal CA-630, with the aim of inactivating the parasites, concentrating the antigenic material, and solubilizing lipophilic proteins such as membrane protein complexes. The success of the inactivation protocol was confirmed by the absence of viable parasites and *T. gondii* DNA after three blind in vitro passages of cell cultures incubated with the vaccine extract, as determined by PCR.

Proteomic characterization by LC‒MS/MS identified a total of 5661 proteins from the vaccine antigens (Additional file 1A) [32]. As expected, proteins derived from host Vero cells (*Chlorocebus sabaeus*) were also identified in the antigen extracts, accounting for 3173 (56%) proteins, whereas 2488 (44%) proteins were identified as originating from *T. gondii*. The quantitative analysis revealed differences in the relative abundance of *T. gondii* proteins between both vaccine antigen preparations, with a higher abundance detected in TgShSp3 than in TgPigSp1 (Additional file 1B). Despite these differences, *T. gondii* proteins from the TgPigSp1 and TgShSp3 vaccine antigens accounted for 92% (2296 out of 2489) of the identified proteins; 6.4% (160) of the identified proteins were exclusively detected for the TgShSp3 vaccine antigen, and 1.3% (33) were detected for the TgPigSp1 vaccine antigen (Additional file 1C). Differential abundance analyses identified 220 proteins with significantly higher levels in TgShSp3 and 46 in TgPigSp1 (adjusted *p* < 0.05; Additional file 1D). When a coefficient of variation threshold < 30% was applied, 128 and 37 proteins remained significantly more abundant in TgShSp3 and TgPigSp1, respectively. Despite these quantitative differences, the immunoblot analysis revealed a largely conserved immunoreactive profile between both antigen preparations, with 23 shared major bands and a limited number of antigen-specific bands (1 and 4 bands exclusive to TgShSp3 and TgPigSp1, respectively) (Additional files 2A and B). These findings suggest minimal differences in overall antigenicity, independent of antigen variations inherent to the genotype. In addition, consistent immunogenicity profiles were observed across different production batches for both isolates, which were subsequently combined for use in vaccine formulations (Additional files 2A and B).

The multistage nature of the vaccine antigens was further assessed by examining the presence of stage-specific proteins associated with tachyzoites and bradyzoites (Additional file 1E). LC‒MS/MS analyses confirmed the presence of these proteins in both antigen preparations, with some bradyzoite markers more abundant in TgShSp3, likely reflecting its greater capacity for bradyzoite differentiation in vitro (Additional file 1E). The presence of the bradyzoite-specific protein TgBAG1 in both the TgPigSp1 and TgShSp3 extracts was confirmed by immunoblotting, supporting the multistage composition of the vaccine candidates (Additional files 2C and D).

### TgShSp3 (Type II-PRU) and TgPigSp1 (Type III) vaccine formulations protected mice from acquired *T. gondii* infection following heterologous challenge with TgShSp1 (Type II-PRU) oocysts

#### T. gondii vaccine formulations were well tolerated and induced a humoral immune response in mice

**Figure 2:**
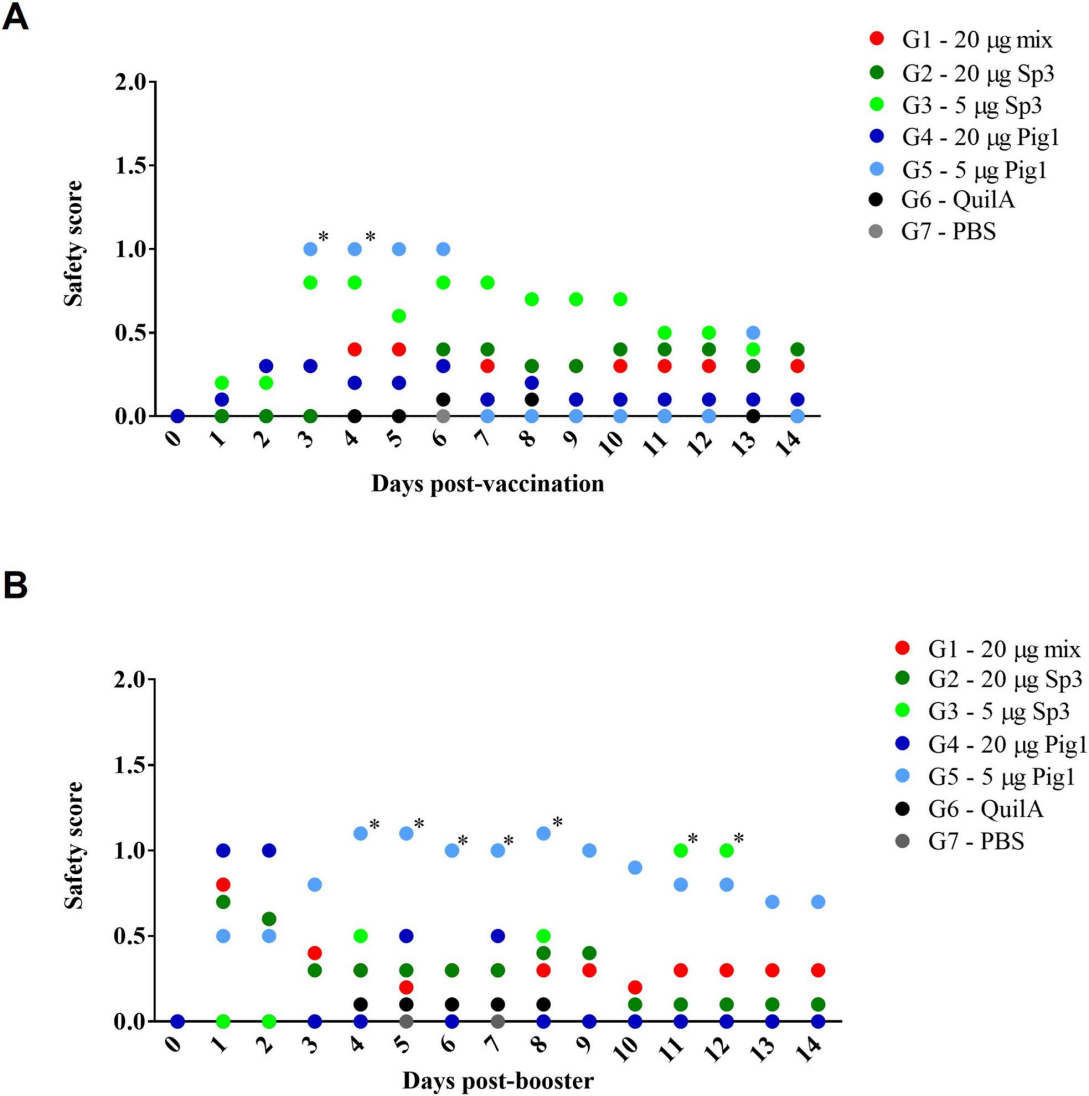
**Clinical monitoring of vaccine safety in mice after vaccination (A) and booster vaccination (B).** Each point on the graph represents the median value according to the established score for adverse effects: systemic (score of 0–4) and local reactions (score of 0–4). Asterisks over each point indicate a significant increase in the adverse effect score of the vaccinated groups compared with that of the G7 group inoculated with PBS, which had an adverse effect score = 0 during all monitoring periods (*p < 0.05; two-way ANOVA). Group G8 inoculated with PBS (score = 0) is not included in the graphs. Note that the y-axis should extend to 8; however, the axis has been adjusted to a maximum value of 2 to improve the clarity and visualization of the graphs, as very mild adverse effects, primarily based on local reactions, were observed.

Two different vaccine formulations for each TgShSp3 and TgPigSp1 antigen, which were based on different antigen quantities (5 µg or 20 µg), were evaluated in a well-established mouse model. The safety profiles of the different TgShSp3 and TgPigSp1 vaccine formulations were evaluated by monitoring systemic side effects (e.g., a ruffled coat, rounded back, and decreased activity) and local reactions following both primary and booster vaccinations (Additional file 3), which demonstrated that the vaccine formulations were highly safe. In all the experimental groups, no relevant systemic side effects were detected (Figure 2), and body weight monitoring revealed no significant differences between the vaccinated and control groups (data not shown). However, a greater number of animals with ruffled coats were observed in the groups vaccinated with 20 µg, particularly after the administration of the booster dose (G1–20 mix: 2 animals (20%) with piloerection after booster, G2–20 Sp3: 4 animals (40%), and G4–20 Pg1: 6 animals (60%) *vs.* G3–5 Sp3 and G5–5 Pg1: with only 1 animal (10%)). One animal from the G1–20 mixed-vaccinated group died suddenly 1 day before the challenge, although no clinical abnormalities were previously detected.

The mice inoculated with 5 µg of the vaccine showed more frequent but mild local reactions that were limited in size (< 5 mm). In some animals, these local reactions developed into small nodules or lesions, which resolved before challenge. Less severe local reactions were also observed in the 20 µg groups (G1–20 mix, G2–20 Sp3, and G4–20 Pig1) on days 3 and 4 post-vaccination (Figure 2A). Similar reactions were observed after the administration of the booster dose to the 20 µg groups, but no significant differences were detected among the vaccinated groups at this point (Figure 2B). However, the 5 µg groups (G3–5 Sp3, G5–5 Pg1) presented significantly more local reactions than did the control groups (G6–Quil-A®, G7–PBS) (*p* < 0.05, two-way ANOVA) (Figure 2B). In some cases, hair loss (alopecia) persisted at the lesion site throughout the trial (G1–20 mix: 4 animals (40%) with nodules/alopecia, G2–20 Sp3: 3 animals (30%), G3–5 Sp3: 7 animals (70%), G4–20 Pg1–2 animals (20%), G5–5 Pg1–7 animals (70%)) (Figures 2A, B). Despite these reactions, the animals recovered well, with hair loss being the only persistent effect. No adverse effects were observed in the control groups (G6–Quil-A®, G7—PBS), ruling out a potential role of the Quil-A® adjuvant in the local reactions.

**Figure 3:**
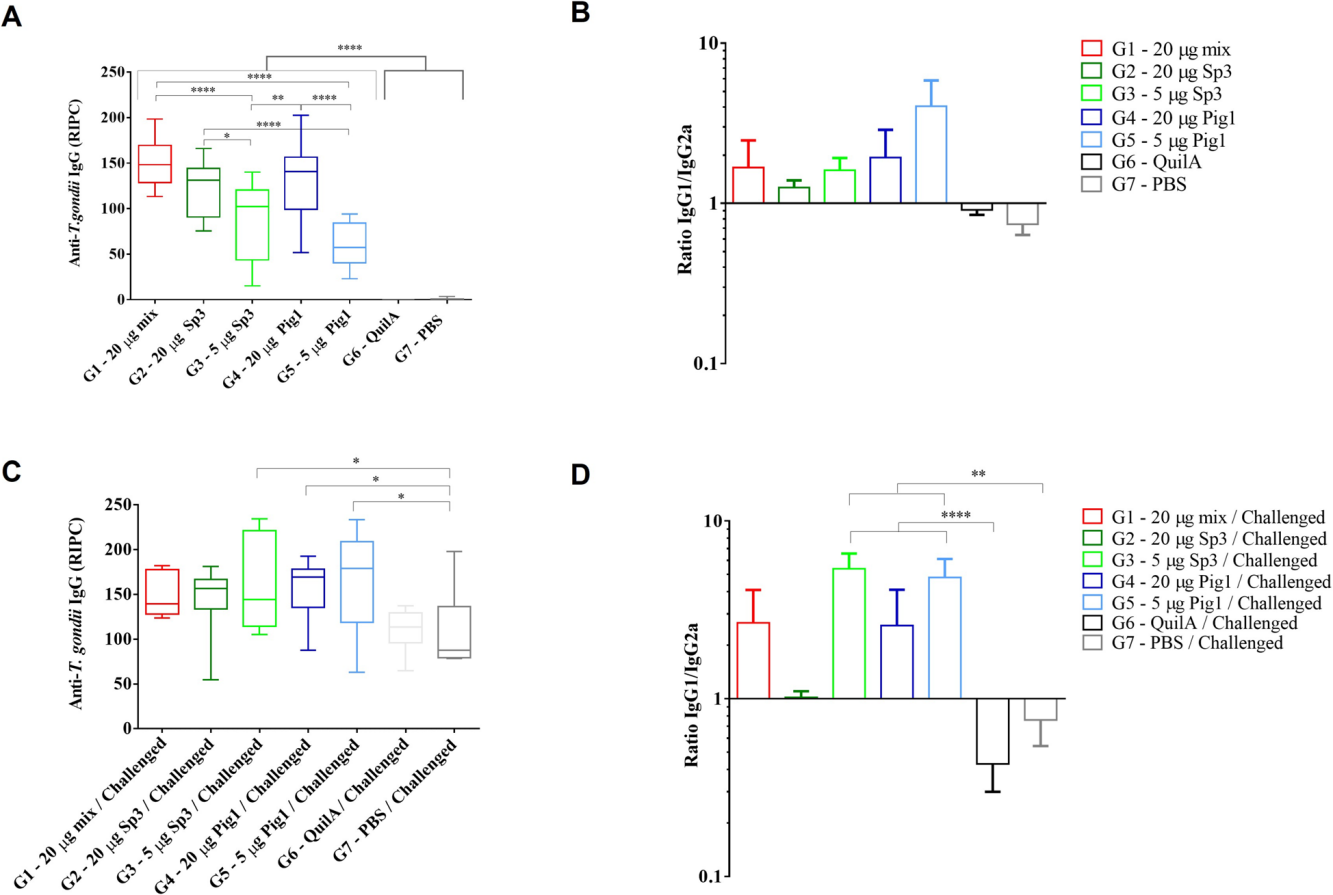
**Levels of IgG antibodies against *T. gondii* after immunization (A, B) and challenge (C, D).** The graphs represent **A** IgG levels after vaccination and booster immunization, measured 5 days prior to the challenge; **B** the ratio of IgG1/IgG2a in the same period; **C** IgG levels after challenge at the end of the experiment; and **D** the ratio of IgG1/IgG2a at this time point. Antibody levels are shown as the relative percent index (RIPC) obtained via an *in-house* ELISA for each group of mice. The horizontal line represents the median; boxes indicate the interquartile range; and whiskers represent the minimum and maximum values. Asterisks above the columns indicate significant differences between the immunized groups and the control groups (G6—Adjuvant and G7—PBS): *p < 0.05; **p < 0.01; ***p < 0.001; and ****p < 0.0001, one-way ANOVA followed by Tukey's multiple comparisons test.

In terms of immunogenicity, mice inoculated with the different vaccine formulations exhibited a significantly stronger immune response against *T. gondii* than the control groups (G6–Quil-A®, G7–PBS). The levels of total IgG, as well as those of IgG1 and IgG2a, were significantly higher in all vaccinated groups than in the G6–Quil-A®, G7–PBS, and G8–Sentinel groups. Additionally, mice vaccinated with 20 µg of the formulation presented higher IgG levels than those vaccinated with 5 µg of the formulation (*p* < 0.05, one-way ANOVA followed by Tukey’s multiple comparisons test) (Figure 3A). Among the vaccinated groups, the G5–5 Pig1 group presented the lowest IgG levels. When the IgG1/IgG2a ratios were evaluated, no significant differences were found among the vaccinated groups, suggesting a balanced Th1/Th2-induced response (Figure 3B).

#### Vaccination reduced the severity of clinical signs and promoted weight maintenance during *T. gondii* infection

**Figure 4:**
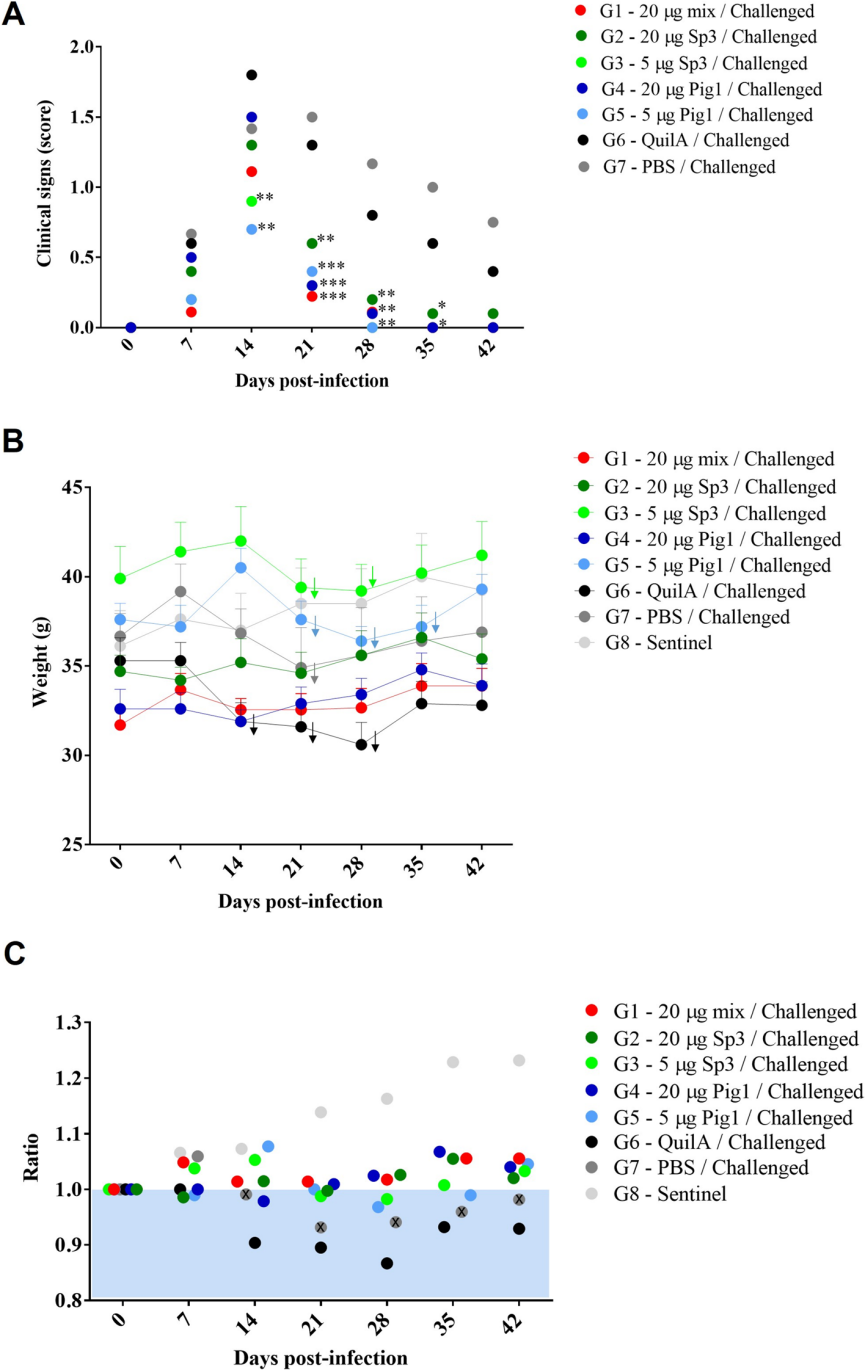
**Morbidity in mice after challenge with TgShSp1 oocysts.** **A** Severity of clinical signs based on the assigned score: ruffled coat (= 1), hunching (= 2), weight loss/body condition deterioration (= 3), and nervous system signs, respiratory signs, or cachexia (= 4). Each point represents the mean value. Asterisks represent significant differences between the vaccinated and control groups (G6—Adjuvant and G7–PBS) (*p < 0.05, **p < 0.01, ***p < 0.001, and ****p < 0.0001; two-way ANOVA). The G8—Sentinel group with no clinical signs (score = 0) is not represented. Note that the y-axis should extend to 4, as the maximum score value, but to improve the clarity and visualization of the graph, it has been adjusted to a value of 2. **B** Weekly weight monitoring throughout the study for all groups. The points represent the mean values, and the bars indicate the standard deviations. The arrows on the right for each point indicate a significant decrease in weight within each group (*p* < 0.05, two-way ANOVA). **C** Ratio of average weight gain from day 0 (before challenge) to day 42 after challenge for each group. Circles with an “X” inside indicate deaths within the group. The shaded blue area indicates groups that remained below threshold 1, reflecting weight loss.

After challenge, the mice were monitored daily for clinical signs associated with *T. gondii* infection (Figure 4). All vaccinated mice survived until day 42 post-infection, whereas two of nine mice (22.2%) in the unvaccinated G7–PBS group died suddenly on days 13 and 16 post-challenge, during the early chronic phase. The mice from all the groups, including the vaccinated and control groups (the G6–QuilA® and G7–PBS groups), presented clinical signs such as ruffled coats and rounded backs during the first 2 weeks post-challenge. On day 14 post-infection, the lowest clinical scores were observed in those groups immunized with 5 µg of the vaccine (G3–5 Sp3, G5–5 Pg1) (*p* < 0.05, two-way ANOVA). Beginning on day 14 post-vaccination, all vaccinated groups were less clinically affected than the unvaccinated groups were, namely, G6 receiving Quil-A® and G7 receiving PBS. During the chronic phase of infection (from 21 days post-infection onward), all the animals across all the groups exhibited ruffled coats. Only a few vaccinated mice displayed a rounded back, whereas most mice in the control groups presented both rounded backs and a loss of body condition (*p* < 0.05, two-way ANOVA) (Figure 4A). Thus, the mice vaccinated with 5 µg (G3–5 Sp3 and G5–5 Pig1) and the unvaccinated control groups (G6–Quil-A® and G7–PBS) experienced significant weight loss starting from the third week post-challenge (Figures 4B and C). In contrast, the body weight of all the mice in the groups vaccinated with 20 µg of the formulations was maintained or increased until the end of the trial, similar to the unchallenged sentinel Group G8, supporting the protective efficacy of the vaccine (Figures 4B and C).

#### Vaccination reduced the parasite load in the target tissues of the mice in a dose-dependent manner

The presence of the parasite in the evaluated target tissues (brain, tongue, quadriceps, and *Longissimus dorsi* muscle) was detected by qPCR in all challenged animals, except for the sentinel group, which tested negative across all tissues.

The detection frequency per organ was lower in the groups immunized with 20 µg of the vaccine (Table 1) than in those receiving 5 µg of the vaccine and the unvaccinated G6—Quil-A® and G7—PBS groups. Specifically, a decrease in the detection frequency of ≥ 20% was observed in the tongue, quadriceps and *Longissimus dorsi* muscles in the groups vaccinated with 20 µg of the formulations compared with those in the G6—Quil-A® and G7—PBS groups. Although the detection frequency in the brain did not vary significantly between groups, the G4–20 Pig1 group presented the greatest reduction in the detection frequency in the brain among all vaccinated groups (Table 1).

**Table 1:** **PCR detection and parasite load reduction in the vaccinated groups versus G7–PBS during the mouse trial**

	TgShSp3 + TgPigSp1 20 μg	TgPigSp1 20 μg	TgShSp3 20 μg	TgPigSp1 5 μg	TgShSp3 5 μg	Adjuvant control	PBS control
Brain							
Detection frequency (%)^a^	100	80	90	90	100	90	100
Mean load reduction (%)^b^	61.9	73	64.1	62	62.3	0	0
Tongue							
Detection frequency (%)	33.3	60	60	80	90	100	100
Mean load reduction (%)	95	96.6	66.4	**0**	**0**	26	0
*Longissimus dorsi* muscle							
Detection frequency (%)	44.4	40	40	90	90	90	100
Mean load reduction (%)	92.3	82.8	89.4	52.3	45.3	**0**	0
Quadriceps muscle							
Detection frequency (%)	55.5	60	80	70	70	100	100
Mean load reduction (%)	97	97.1	92.1	15.8	24.1	3.3	0
Total							
Detection frequency (%)	58.2^1^	60^1^	67.5^1^	82.5^1^	87.5^1^	95	100
Mean load reduction (%)	81.9^2^	84.5^2^	71.2^2^	57.8	58.6	**0**	0

^a^Number of positive animals/total animals in the group.

^b^percentage of parasite load reduction compared with the control group. Parasite loads were higher than those of the PBS control group; therefore, no reduction in parasite load was observed and is indicated in bold type.

^1^Significant differences compared with the PBS control group (*p* < 0.001 Fisher’s exact test).

^2^Significant differences compared with the PBS control group (*p* < 0.0001, Mann– Whitney test).

**Figure 5:**
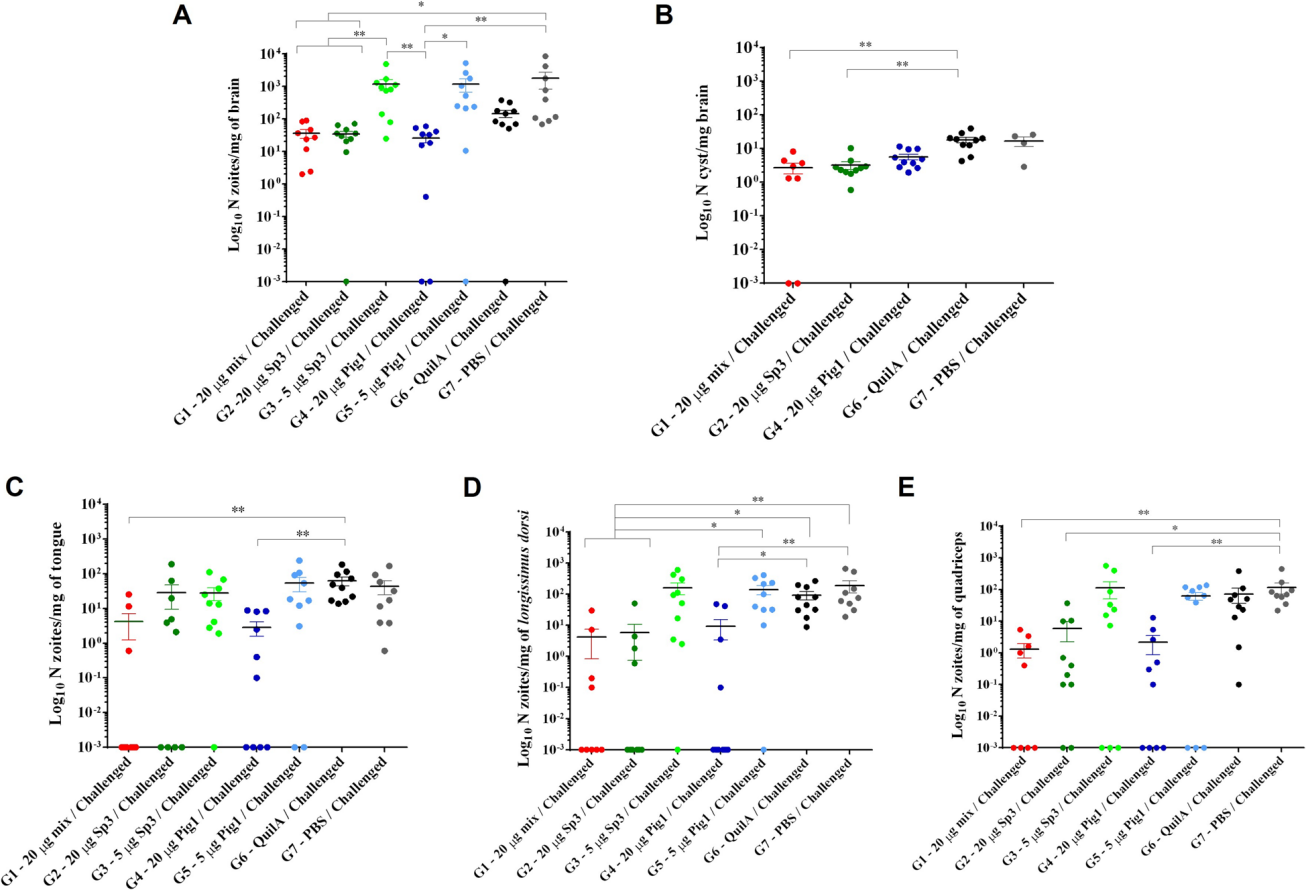
**Parasite load quantification and brain cyst count in the mouse trial.** The graphs show parasite loads quantified in the brain (**A**), tongue (**C**), *Longissimus dorsi* muscle (**D**) and quadriceps muscle (**E**), as well as brain cyst count (**B**). Each point in the graphs represents the number of zoites per mg of tissue or number of cysts per mg of brain detected in each mouse from the different immunized groups (G1–G5) and challenged control groups (G6—Adjuvant and G7—PBS). The horizontal line indicates the mean values and the error bars represent the SEM. Asterisks above the columns denote significant differences between groups (*p < 0.05 and **p < 0.01, Kruskal‒Wallis test followed by Dunn’s multiple comparisons test). Parasite loads of 0 are represented in the graphs with a value of 0.001 according to the logarithmic scale of the graph. The G8–Sentinel group with a load of 0 has not been included in the graphs.

The analysis of the parasite load detected per organ is presented in Figures 5A, C, D, E. Importantly, the reduction in the mean parasite load in the vaccinated groups, compared with that in the G7–PBS group, was greater than 70% in all the tissues of the animals immunized with 20 µg of the vaccine, along with a nearly 40% reduction in the parasite detection frequency (Table 1). Specifically, in the G4–20 Pig1 group, all the tissues presented a significant reduction in the parasite load of more than 82%, with a detection frequency of less than 60% in all the samples, except in the brain, where the load reduction was 73% and the detection frequency was 80%. In the other groups that received 20 µg of the vaccines (G1–20 mix and G2–20 Sp3), the reduction in parasite load was greater in the *Longissimus dorsi* muscle but lower in the other organs. In general, most animals vaccinated with 20 µg showed parasite loads between 0 and 10 zoites, whereas the other groups presented loads ranging from 10 to 100, with an observed reduction of approximately tenfold when comparing groups, except in the brain, where the reduction was smaller, with parasite loads nearly 5 times lower than those of the control group. No significant differences were observed between the loads detected in the groups vaccinated with 5 µg of the formulations and the unvaccinated challenged control groups in the different target organs (Figure 5). In these groups, the reductions in the mean parasite loads and detection frequencies in the tissues were minimal compared with those in the control group, G7, which received PBS. In some organs, the mean parasite loads in the groups vaccinated with 5 µg of the formulations were even higher than those in the control group (Table 1). Specifically, in the brain, significant differences were observed between the groups immunized with 20 µg of the vaccines (G1–20 mix, G2–20 Sp3, and G4–20 Pig1) and the groups that received 5 µg of the vaccines (G3–5 Sp3 and G5–5 Pig1), which presented the highest parasite loads, as did the G7–PBS group (*p* < 0.05, Kruskal‒Wallis test followed by Dunn’s multiple comparisons test) (Figure 5A). In the tongue, significant differences were observed between the G1–20 mix and G4–20 Pig1 groups, which presented the lowest loads, compared with the G6–Quil-A® group (*p* < 0.05, Kruskal‒Wallis test followed by Dunn's multiple comparisons test) (Figure 5C). In the quadriceps and *Longissimus dorsi* muscles, all the groups inoculated with 20 µg of the vaccines presented significantly lower parasite loads than did the G7–PBS group (Figures 5D, E).

*T. gondii* brain cysts were also counted in the groups immunized with 20 µg of the vaccines because of their greater efficacy in the previous analysis and in the G6–Quil-A® and G7–PBS control groups. The results showed similar differences in the parasite loads detected by qPCR between the vaccinated and unvaccinated groups. The number of cysts in the groups inoculated with 20 µg of mixed (G1–20 mix) and TgShSp3 (G2–20 Sp3) vaccine formulations was significantly lower than that in the G6–Quil-A® group (Figure 5B) (*p* < 0.05, Kruskal‒Wallis test followed by Dunn’s multiple comparisons test).

#### Vaccination induced a strong humoral immune response in mice after the challenge

All challenged mice presented high IgG levels against *T. gondii* infection (Figure 3C). No significant differences were observed between the vaccinated groups after the challenge. Nevertheless, the mice vaccinated with 5 µg of the vaccines (G3–5 Sp3 and G5–5 Pig1) presented significantly higher IgG levels together with higher IgG1/IgG2a ratios than did the challenged control groups (G6–Quil-A® and G7–PBS) (*p* < 0.05, one-way ANOVA followed by Tukey’s multiple comparisons test) (Figures 3C and D). These findings reflect a greater IgG1 response in the 5 µg-vaccinated groups (G3–5 Sp3 and G5–5 Pig1), which was likely associated with a predominance of a Th2 response. In fact, all the groups inoculated with the different vaccine formulations presented significantly higher IgG1 levels than did the adjuvant control group, and no significant differences were detected in the IgG2a levels between the vaccinated and control groups.

### Vaccination in piglets was safe and very effective at limiting *T. gondii* infection in target tissues

Vaccine formulations based on 20 µg and 40 µg of the TgPigSp1 (Type III) antigen were produced and evaluated for their ability to prevent *T. gondii* infection in piglets via heterologous challenge with 1000 oocysts of the TgShSp1 (Type II-PRU) isolate.

#### The TgPigSp1 (Type III) vaccine formulation was highly safe in piglets

No systemic adverse effects were observed in the piglets after vaccination or booster treatment. Adverse effects following immunization were limited to occasional episodes of diarrhea lasting no more than 1 day in all piglet groups involved in the vaccine trial, which was likely associated with gradual dietary changes after weaning. One case of pododermatitis was detected in one animal from the G4–Sentinel group, which was treated with antibiotic and anti-inflammatory medications and resolved satisfactorily within a few days. All these adverse effects could not be directly associated with the vaccination.

**Figure 6:**
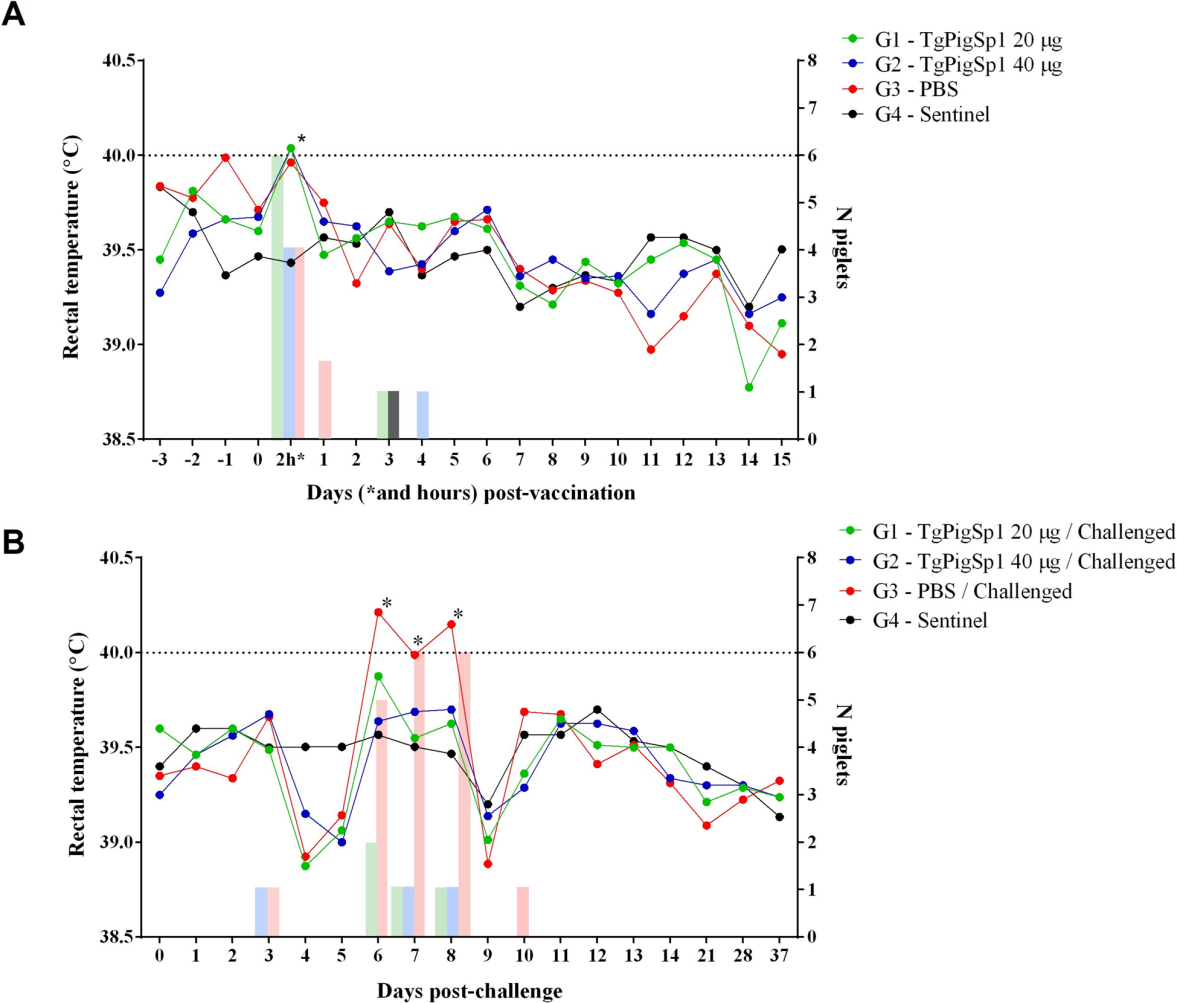
**Mean rectal temperatures of each piglet post-vaccination (A) and post-challenge (B).** The horizontal dashed line indicates the hyperthermia threshold, while the bars represent the number of animals that developed a rectal temperature > 40 °C on the days after vaccination/challenge. Asterisks indicate differences in rectal temperature compared with the G4—Sentinel group (* *p* < 0.05, two-way ANOVA).

Rectal temperature monitoring detected an increase (≥ 40 °C) in six animals (75%) from G1–20, four animals (50%) from G2–40, and four animals (50%) from G3–PBS at 2 h post-immunization, likely due to handling during vaccination. During the 15-day follow-up period after immunization, sporadic hyperthermia (≥ 40 °C) was detected in no more than two animals in Groups G1–20, G2–40, G3–PBS, and G4–Sentinel, with episodes lasting no more than 1 day. No significant differences in average rectal temperature were observed between the groups inoculated with the different vaccine formulations and the G3–PBS and G4–Sentinel groups (Figure 6A). The average temperature increase in any group during the follow-up period was less than 1 °C.

No local reactions, including pain, swelling or redness around the site of injection, were observed in any group after the intramuscular vaccination or booster with the vaccine prototype. Thus, the vaccine formulations were considered very safe.

#### The TgPigSp1 vaccine formulation elicited high anti-*T. gondii* IgG and IFN-γ levels prior to challenge

**Figure 7:**
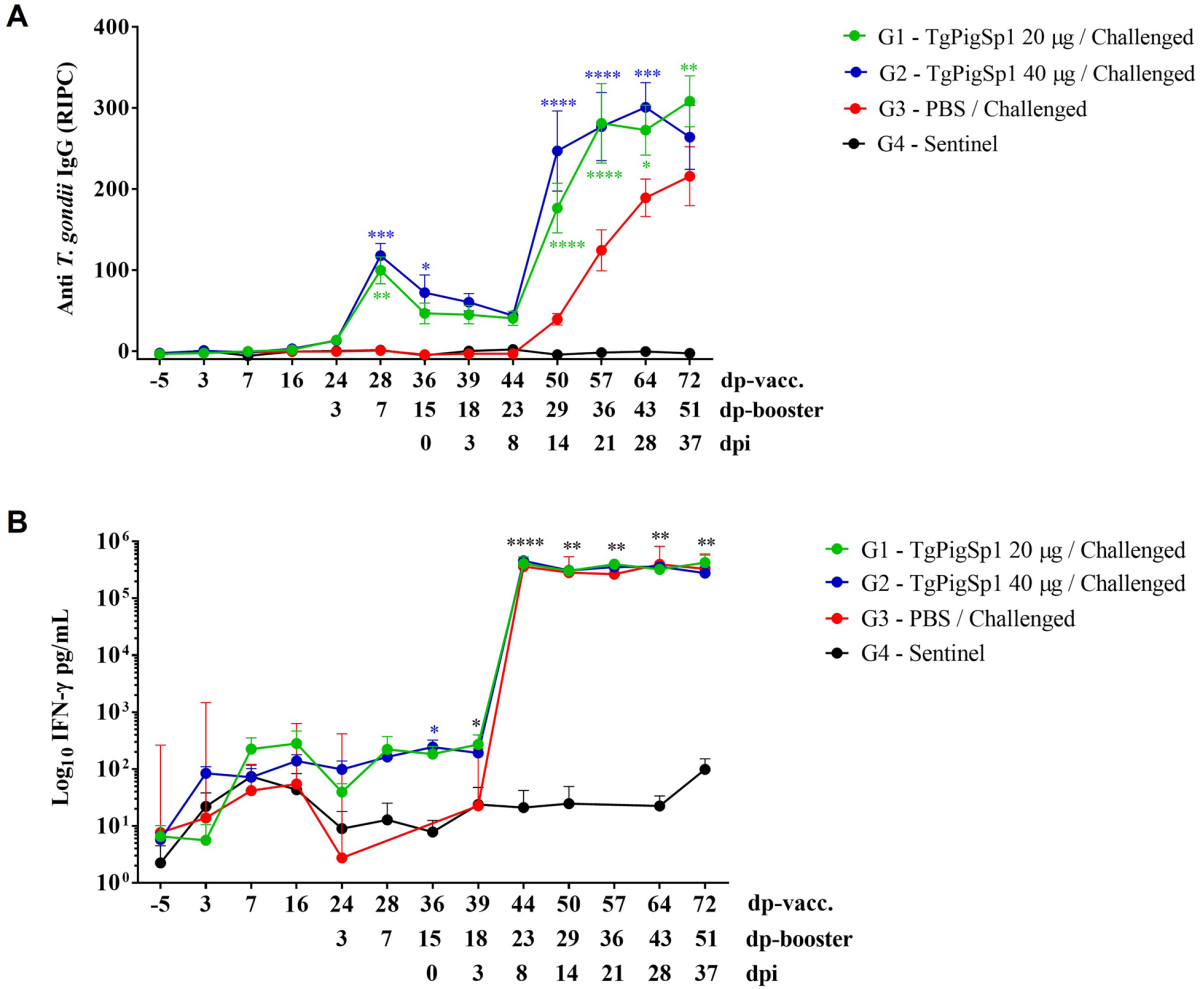
**Levels of anti-*T. gondii* IgG (A) and IFN-γ production (B) throughout the piglet trial.** The anti-*T. gondii* IgG levels are reported as the relative index percent (RIPC) obtained from an in-house ELISA. The vertical bars indicate the standard deviation. Asterisks above the data points denote significant differences between the immunized groups and the G3–PBS group: *p < 0.05, **p < 0.01, ***p < 0.001, and ****p < 0.0001; two-way ANOVA. **B** The mean IFN-γ level in pg/mL for each group. The vertical bars indicate the standard deviations. Asterisks above the data points denote significant differences between the challenged groups (G1–20, G2–40 and G3–PBS) and the G4–Sentinel group: * *p* < 0.05, **p < 0.01, and **** *p* < 0.0001; two-way ANOVA.

Cellular and humoral immune responses induced by different vaccine formulations were evaluated by measuring the amount of IFN-γ produced upon antigen stimulation in vitro and the levels of anti-*T. gondii* IgG, IgG1, and IgG2a in the serum after vaccination and booster immunization. After the first vaccination, no changes in antibody levels were observed in the vaccinated animals (Figure 7A, Additional files 4A and B). A significant increase in IgG levels was detected 7 days after the booster immunization in the vaccinated groups, G1–20 and G2–40 (Figure 7A), with similar kinetics observed for the IgG1 and IgG2a isotypes (Additional file 4A and B). Therefore, the analysis of IgG, IgG1, and IgG2a levels produced in response to *T. gondii* revealed significantly higher antibody levels in the vaccinated groups than in the control group on day 7 post-booster, which remained higher until challenge (*p* < 0.05; two-way ANOVA). No significant differences were found in the IgG1/IgG2a ratio between the vaccinated groups (*p* > *0.05*; two-way ANOVA) (Additional file 4C).

The analysis of *T. gondii-*specific IFN-γ production in supernatants obtained from in vitro blood cell cultures stimulated with *T. gondii* antigen following vaccination and booster immunization is shown in Figure 7B. IFN-γ levels did not increase significantly in any of the evaluated groups prior to challenge. However, the group immunized with 40 µg of the vaccine prototype (G2–40) presented significantly higher IFN-γ levels on days 15 and 18 after the booster, corresponding to days 0 and 3 post-challenge, respectively, than did the control group, indicating an early cellular response to infection (*p* < 0.05; two-way ANOVA).

#### Clinical signs were milder in vaccinated piglets following *T. gondii* infection

No severe clinical consequences attributable to oocyst infection were observed in the challenged animals. Specifically, no diarrhea or other notable systemic clinical signs were recorded in any of the challenged piglets. Only animals in the unvaccinated control group (G3–PBS) exhibited transient lethargy and a reduction in appetite for the first week post-challenge, as evidenced by lower food consumption between days 6 and 8 post-infection. These 3 days coincided with the hyperthermia (≥ 40 °C) recorded in all the animals in the G3—PBS group, which presented significantly higher temperatures than the vaccinated animals did (*p* < 0.05, two-way ANOVA) (Figure 6B). In the vaccinated groups, sporadic hyperthermia was recorded in only three animals (37.5%) from group G1–20 and one animal (12.5%) from group G2–40, each for a single day during the same time frame, without any other clinical signs, such as behavioral changes (Figure 6B).

#### Vaccination enhanced and prolonged the immune response after challenge

Seropositive IgG levels in the vaccinated groups (G1–20 and G2–40) were maintained until day 8 post-challenge, after which they increased significantly and remained elevated until the end of the trial. Thereafter, the IgG levels in the vaccinated groups were significantly higher than those in the unvaccinated challenged group (G3–PBS) (*p* < 0.05; two-way ANOVA) (Figure 7A). In the G3–PBS control group, seroconversion became significant only on day 21 post-challenge (*p* < 0.05; two-way ANOVA). The sentinel group (G4—Sentinel) remained at baseline levels throughout the study.

Similarly, the IgG1 and IgG2 levels in the vaccinated groups, G1–20 and G2–40, were maintained until day 14 post-infection, after which they increased significantly and remained elevated until the end of the experiment (Additional files 4A and B). Beginning on day 14 post-infection, the levels in the immunized groups were significantly higher than those in the G3–PBS and G4–Sentinel groups, without significant differences between the vaccinated groups (*p* < 0.05; two-way ANOVA) (Additional files 4A and B). At the time of seroconversion, no significant differences were detected in the IgG1/IgG2a ratios among the animal groups (Additional file 4C).

The IFN-γ levels were significantly increased in all the challenged groups on day 8 post-challenge and remained elevated until the end of the trial (Figure 7B). However, no significant differences were detected between the immunized groups (G1–20 and G2–40) and the challenged control group (G3–PBS) throughout the trial (Figure 7B).

#### Vaccination reduced the parasite burden in target tissues

The efficacy of the different vaccine formulations was evaluated through an analysis of the detection frequency using a mouse bioassay, the “gold standard” technique for determining *T. gondii* viability and infectivity of tissue cysts in infected tissues. The percentage of *T. gondii* detected among the groups using this technique was consistently higher in the unvaccinated G3–PBS group (Table 2). The vaccinated groups presented a 62.5% reduction in the number of infected piglets in G1–20 (5 out of 8 *T. gondii*-free piglets) and 50% in G2–40 (4 out of 8 *T. gondii*-free piglets) compared with 100% detection in G3–PBS. Detection frequencies in porcine target tissues were significantly lower in the vaccinated groups, with no more than two *T. gondii*-positive samples identified among all assayed tissues (≤ 16.6% of assayed tissue samples for each piglet; Table 2), whereas the frequencies were higher in most tissues of G3—PBS animals (≥ 25% of assayed tissue samples for each piglet; Table 2). Therefore, the detection frequency per group was reduced by more than 88% in both vaccinated groups (G1–20: 90.5%; G2–40: 88.1%) compared with the G3—PBS group. The tissue-specific analysis revealed significant reductions among the different groups, with detection frequencies decreasing by more than 83% in the brain and heart and by more than 85% in the *Longissimus dorsi* and semimembranosus muscles of the vaccinated groups compared with those of the G3–PBS group (Table 2). Additional detection analyses based on ITS-1 PCR and qPCR targeting the 529-RE region showed limited detection in the vaccinated groups G1–20 and G2–40 compared with the G3—PBS group. A detailed summary of *T. gondii* detection in target tissues for each piglet from all groups is provided in Table 2.

**Table 2:** **Summary of results on parasite detection in mouse bioassay, and direct nested-PCR and qPCR of pig tissue extracts from brain, heart, loin, and semitendinosus muscle samples from each animal**

	Brain		Heart		*Longissimus dorsi*		Semimembranosus			
	Mouse Bioassay PCR/serology (IgG-G-or IgM-M-)	Nested-PCR (qPCR-N zoites/mg extract)	Mouse Bioassay PCR/serology (IgG-G-or IgM-M-)	Nested-PCR (qPCR -N zoites/mg extract)	Mouse Bioassay PCR/serology (IgG-G-or IgM-M-)	Nested-PCR (qPCR-N zoites/mg extract)	Mouse bioassay PCR/serology (IgG-G-or IgM-M-)	Nested-PCR (qPCR-N zoites/mg extract)	Detection frequency per animal by mouse bioassay: + /n (%)	Detection frequency per animal by nested-PCR: + /n (%)
G1 (Vaccine TgPigSp1—20 μg)										
1	− − −/− − −	− −	− − −/− − −	− −	− − −/− − −	− −	− − −/− − −	+ +	0/12 (0)	2/8 (25)
2	− − −/− − −	− −	− − −/− − −	− −	− − −/− − −	− −	− − −/− − −	− −	0/12 (0)	0/8 (0)
3	− − −/− − −	− −	− − −/− − −	+ +	− − +/− − G	− −	− − −/− − −	− +	1/12 (8.3)	3/8 (37.5)
4	− + −/− G −	+ +	− − −/− − −	− −	− − −/− − −	− −	− − −/− − −	− −	1/12(8.3)	2/8 (25)
5	− + −/− G −	+ + (0.115)	− − −/− − −	+ + (0.204)	− − −/− − −	+ +	− − +/− − G	+ +	2/12 (16.6)	8/8 (100)
6	− − −/− − −	− +	− − −/− − −	− −	− − −/− − −	− +	− − −/− − −	− −	0/12 (0)	2/8 (25)
7	− − −/− − −	− +	− − −/− − −	− −	− − −/− − −	− +	− − −/− − −	− −	0/12 (0)	2/8 (25)
8	− − −/− − −	− −	− − −/− − −	− −	− − −/− − −	+ +	− − −/− − −	− −	0/12 (0)	2/8 (25)
Detection frequency: + /n (%)	2/24 (8.3)^1^	6/16 (37.5)^1, a^	0/24 (0)^1^	6/16 (37.5)^2^	1/24 (4.1)^1^	6/16 (37.5)^2^	1/24 (4.1)^1^	5/16 (31.2)^1^	Total: 4/96 (4.16)^1^	Total: 21/64 (32.8)^1^
G2 (Vaccine TgPigSp1 − 40 μg)										
9	− − −/− − −	− +	− − −/− − −	− −	− − −/− − −	− −	− − −/− − −	− −	0/12 (0)	1/8 (12.5)
10	− + −/− G −	+ −	− − −/− − −	+ +	− − −/− − −	− −	− − −/− − −	+ −	1/12 (8.3)	4/8 (50)
11	− − −/− − −	− −	− − −/− − −	− −	− − −/− − −	− −	− − −/− − −	+ −	0/12 (0)	1/8 (12.5)
12	− − −/− − −	+ +	− − −/− − −	− −	− − −/− − −	− −	− − −/− − −	− +	0/12 (0)	3/8 (37.5)
13	− − −/− − −	− −	+ − − /G − −	+ +	− − −/− − −	− −	− − −/− − −	− −	1/12 (8.3)	2/8 (25)
14	− − −/− − −	− −	− − −/− − −	− −	− − −/− − −	− −	− − −/− − −	− −	0/12 (0)	0/8 (0)
15	− − −/− − −	− −	+ − − /G − −	− −	+ − − /G − −	+ − (0.115)	− − −/− − −	+ +	2/12 (16.6)	3/8 (37.5)
16	− − −/− − −	+ −	− − −/− − −	− −	− − −/− − −	+ +	+ − − /G − −	+ +	1/12 (8.3)	5/8 (8.3)
Detection frequency: + /n (%)	1/24 (4.1)^1^	5/16 (31.2)^1, a^	2/24 (8.3)^1^	4/16 (25)^1, a^	1/24 (4.1)^1^	3/16 (18.75)^1^	1/24 (4.1)^1^	7/16 (43.75)	Total: 5/96 (5.2)^1^	Total: 19/64 (29.6)^1^
G3 (Unvaccineted/Challenged)										
17	− − −/− − −	+ + (0.508)	− − +/− − G	+ +	− − −/− − −	− −	− − −/− − −	− +	1/12 (8.3)	5/8 (62.5)
18	+ + − /G G −	+ +	+ + + /G G G	+ + (1.358)	− + +/− G G	+ + (12.123)	− + −/− G −	+ +	8/12 (66.6)	8/8 (100)
19	+ − + /G − G	+ + (14.587)	+ + + /G G G	+ + (3.929)	+ + − /G G −	+ + (0.380)	+ + − /G G −	+ +	9/12 (75)	8/8 (100)
20	+ − − /G − −	+ +	+ − + /G − G	+ +	− − −/− − −	− −	− − −/− − −	− −	3/12 (25)	4/8 (50)
21	+ − − /G − −	− + (0.194)	+ − − /G − −	+ + (0.494)	− − −/− − −	− −	+ − − /M − −	− −	3/12 (25)	3/8 (37.5)
22	+ − − /G − −	+ + (0.804)	+ − + /G − G	+ + (0.736)	+ − + /G − G	+ +	− − −/− − −	+ +	5/12 (41.6)	8/8 (100)
23	+ − + /G − G	+ + (0.168)	+ + + /G G G	+ + (0.356)	+ + − /M G −	+ +	+ + + /G G G	+ +	10/12 (83.3)	8/8 (100)
24	+ + + /M G G	+ + (0.549)	− − −/− − −	− −	− − −/− − −	− −	− − −/− − −	− −	3/12 (25)	2/8 (25)
Detection frequency: + /n (%)	12/24 (50)	15/16 (93.75)	14/24 (58.3)	14/16 (87.5)	9/24 (37.5)	8/16 (50)	7/24 (29.1)	9/16 (56.2)	Total: 42/96 (43.75)	Total: 46/64 (71.8)

G; parasite detection uniquely by IgG serology; M; parasite detection uniquely by IgM serology.

^1^Significant differences vs. the G3 group and 2 vs. G2 group (*p* < 0.05, Fisher’s exact test).

^a^Significant differences vs. the G3 group (*p* < 0.05, Kruskal‒Wallis test followed by Dunn’s multiple comparisons test).

By qPCR quantification, only two samples from the same pig (brain and heart) were positive in the G1–20 group. Only one *Longissimus dorsi* tissue sample was positive in the G2–40 group (Table 2), whereas parasites were quantified in the brain, heart, and loin of 7 of 8 piglets in the G3—PBS group (Table 2). Significant reductions in the parasite load were observed in the brain (Table 2) for both vaccinated groups (G1–20 and G2–40) and in the heart (Table 2) for group G2–40 compared with the challenged control group G3—PBS (*p* < 0.05, Kruskal‒Wallis test followed by Dunn’s multiple comparison test). These limited parasite loads may explain the reduced detection frequencies in target tissues of piglets from the vaccinated groups.

The detection of *T. gondii* using the more sensitive ITS-1 PCR also revealed a reduction of more than 50% in both vaccinated groups (G1–20: 54%; G2–40: 58%) compared with the G3—PBS group (100%) (Table 2). Specifically, detection in the brain was reduced by more than 60% in both vaccinated groups, by more than 70% in the heart, and by more than 60% in the *Longissimus dorsi* muscle of the G2–40 group, supporting the lower detection rate in the mouse bioassay.

Potential associations between the vaccine-induced immune response and parasite detection in piglet target tissues were also individually assessed (Additional file 5). In the G1‒20 group (Additional files 5A and C), piglet 5 presented the lowest IgG and IFN-γ levels throughout the study until challenge and presented the highest detection rates according to bioassays (16.6%) (Table 2) and nested PCR (100%) (Table 2). This result may indicate insufficient immune stimulation and reduced protection. However, no notable associations were observed in the G2–40 group at this stage (Additional files 5B and D).

## Discussion

In this study, we evaluated a novel multistage inactivated vaccine derived from a recently obtained *T. gondii* isolate and showed its safety and efficacy against acquired toxoplasmosis in piglets. These promising results support its potential implementation as a preventive measure to reduce the zoonotic transmission of *T. gondii* to humans. Recent investigations have shown that clonal genotype II is the most prevalent genotype among *T. gondii* isolates circulating in Western Europe, followed by genotype III, both of which have been identified in domestic and wild animals [33]. Notably, these isolates retained their ability to spontaneously form fully developed tissue cysts in vitro, as confirmed by the proteomic analysis in this study [18]. The field isolates TgPigSp1 and TgShSp3, which are maintained under limited in vitro passages, could thus preserve antigenic profiles more representative of *T. gondii* strains circulating in natural infections than laboratory-adapted strains commonly used in previous studies, which may have undergone drift due to prolonged culture adaptation [18, 34].

A vaccine targeting both the tachyzoite and bradyzoite stages, representing the acute and chronic phases of infection, has long been considered a more effective approach to limit the chronic persistence of infection in intermediate hosts [17]. While this strategy has been extensively explored in recombinant or DNA vaccine platforms, its efficacy remains limited [15]. In addition to the need for multistage targeting, a recent study revealed CD8⁺ T-cell responses against the latent form of *T. gondii*, highlighting the role of the cyst as a replicative reservoir that mitigates host tissue damage and ensures parasite persistence [35]. However, the number of antigens available for recombinant platforms is limited, and their immunogenicity may vary depending on the expression system. Therefore, the use of native antigens obtained directly from tachyzoites and bradyzoites of field isolates, as pursued here, provides a promising alternative. Moreover, although the experimental challenge in this study used oocysts, natural infection in pigs can also occur through the ingestion of tissue cysts [1]. Therefore, the presence of bradyzoite antigens in the vaccine may confer an added advantage in controlling infection via this route.

The inactivated vaccine was designed to eliminate the biosafety risks associated with live vaccines, both for animals and handlers, while improving stability for storage and distribution. Additionally, the antigens were produced in Vero cells, a standard substrate for industrial vaccine production, enabling scalable and consistent antigen yields [36]. The antigen processing protocol was also designed for the concentration and solubilization of parasite components involved in protein complexes from the membrane, which were combined with *Quillaja saponaria* saponin [37–39], a veterinary-grade adjuvant known to induce a balanced Th1/Th2 immune response critical for the control of *T. gondii* [40].

The safety, immunogenicity, and efficacy of the vaccine were first evaluated in a murine model and then validated in piglets, the target host species. The murine model is widely used in the development of *T. gondii* vaccines because of its low cost, ease of handling, and availability of advanced immunological tools to assess immune responses [41]. Additionally, the high susceptibility of mice to *T. gondii* infection allows for a detailed evaluation of key parameters, such as reductions in the parasite burden and tissue cyst formation, providing essential insights into screening the protective capacities of vaccines [29]. Nevertheless, the virulence of *T. gondii* isolates can vary depending on the host species used for challenge [22]. As a method to address this issue, standardized infection models previously implemented and validated in mice and piglets were employed to ensure robust, comparable efficacy assessments across species [21, 23]. Despite their anatomical and immunological differences, both models presented consistent infection patterns, enhancing the translational relevance of the results from murine models to swine. In fact, the *T. gondii* infection models used in this study showed comparable clinical and parasite tissue distributions of TgShSp1 (Type II-PRU) and TgShSp24 (Type III) isolates for chronic infection, enhancing the translational value of the immunity and efficacy of vaccines between both species, as observed in this study [23].

All vaccine formulations tested in mice were generally well tolerated, with no significant systemic adverse effects observed compared with the control groups. Local reactions were mild and transient, although more frequent in the groups that received the lower antigen dose. These results indicated that although the vaccines are safe, the 20 µg dose appears to be more suitable. A high tolerance profile to vaccination was observed in piglets, which is likely the most sensible category, as no systemic adverse effects or local reactions were observed following vaccination or the booster dose. In this study, potential adverse effects were not related to vaccination since a slight increase in temperature was recorded in some animals across all groups a few hours after inoculation, regardless of whether the vaccine or PBS was administered, and it was associated with stress induced by the handling procedure. Consistent with previous studies evaluating live and inactivated vaccines, no increase in body temperature was detected during the 14 days post-vaccination [14, 42]. These findings advance the development of inactivated vaccines as safer alternatives for target species.

In this study, mice were vaccinated with a relatively high dose independent of the origin of the vaccine antigen, i.e., 20 µg exhibited the best efficacy and a more consistent reduction in parasite burden across key tissues, including the brain, muscles, and tongue. The amount of antigen in vaccine formulations is crucial for efficacy, especially in inactivated vaccines, because it should be suitable to induce sufficient immune stimulation at the levels required to limit infection. Unlike live vaccines, double–triplet stimulation is required to obtain the desirable efficacy [15]. However, in addition to the capacity of the adjuvant used for immune stimulation, the amount of the antigen could influence efficacy, as was observed in previous studies with recombinant rTgROP17 [43]. In this study, although the 5 µg dose induced an immune response, it was less effective at limiting infection than the 20 µg dose. Mice vaccinated with the higher dose presented elevated IgG levels, along with a more favorable IgG1 to IgG2a ratio, with particularly high IgG2a (Th1) levels. This Th1-dominant response is crucial for attenuating *T. gondii* infection, as it promotes the activation of immune cells that restrict parasite replication within host cells. While some level of the Th2 response (associated with IgG1) may contribute to immune protection, the predominance of Th1 responses is key to controlling *T. gondii*, particularly for effective intracellular parasite control in mice [44–46].

Importantly, no relevant differences in immunogenicity or efficacy were observed in mice, depending on the isolate used to obtain the vaccine antigen, despite the potential differences in antigenicity due to genotype (Type II-PRU vs. Type III). Similarly, the real *T. gondii* antigen dose in the vaccine formulation, as revealed by vaccine antigen characterization, did not significantly affect the immune response or the achievement of optimal efficacy. Nevertheless, although the difference was not statistically significant, a greater efficacy was observed for 20 µg of the TgPigSp1 vaccine formulation in terms of the reductions in the frequency and load in brain, tongue and quadriceps muscle compared with the same dose of the TgShSp3 vaccine. Heterologous protection was confirmed with a Type III (TgPigSp1) vaccine antigen against Type II-PRU (TgShSp1) infection, the predominant genotype in the Northern Hemisphere [33]. Thus, two doses of the TgPigSp1 vaccine antigen were selected for evaluation in the piglet trial against heterologous challenge with oocysts of the TgShSp1 isolate.

The first clues concerning the efficacy of the vaccine in piglets were reductions in both the occurrence and severity of clinical signs in vaccinated animals, with only a limited number of animals experiencing mild hyperthermia for a shorter period. In contrast, in the unvaccinated group, all the animals developed hyperthermia during the first days after challenge, along with anorexia and prostration, as previously described in the experimental piglet model [21, 47–49]. Therefore, this lower severity of clinical signs among vaccinated animals could be an initial indication that the infection is better controlled.

This study provides strong evidence that vaccination with the inactivated TgPigSp1 parasite vaccine, followed by challenge with TgShSp1 oocysts in pigs, effectively and significantly reduces the establishment of viable tissue cysts, as indicated by the results of the mouse bioassay. Infection rates were markedly different between mice inoculated with tissues from pigs in Groups G1–20 and G2–40 compared with those inoculated with tissues from pigs in the G3—PBS control group. Specifically, the reduction in the detection frequency exceeded 88–90% in vaccinated animals.

Among the evaluated tissues, the brain and heart were selected because of the preference of *T. gondii* for these organs, given their central nervous system location and large blood supply. The muscles (*Longissimus dorsi* and semimembranosus) were chosen for their commercial relevance in terms of human consumption [21]. In vaccinated animals, the detection frequency was significantly reduced at similar levels across all evaluated tissues. The median infectious dose 50 (ID_50_) for the TgShSp1 isolate was previously established at approximately 11 tachyzoites [50], which aligns with the parasite loads determined in the target piglet tissues in this study, which were > 11 zoites per mg in a very limited number of samples. In the positive muscle samples detected in the vaccinated pigs, the parasite loads were at least 35 times lower than the mean load in the unvaccinated pigs. These results indicate a substantial reduction in the risk of zoonotic transmission. The results from ITS1 PCR revealed higher detection frequencies, with reductions of over 54–58%. However, the detection limit of nested PCR can reach a single zoite, which, according to previous studies, is not infectious in mice for this isolate [50]. In addition, these findings remain significant given that ITS1 PCR can be used to identify parasite DNA, which may be derived from nonviable parasites persisting in the tissue before being cleared by immune responses.

A limited number of studies have evaluated the efficacy of inactivated and killed vaccines against *T. gondii* infection in pigs and even in mice [15, 39]. Most of the previous studies assessed recombinant and DNA vaccines with only partial efficacy against lethal acute and chronic infections in mice [15]. The first studies in mice with killed tachyzoites alone or with adjuvants offered only slight protection against challenge with the M-7741 isolate and no protection against challenge doses that were lethal [42]. Neither liposomes nor lauric or myristic anhydride alone or potentiated with levamisole surpassed Freund's adjuvant in producing slight but inconsistent immunity in mice [51]. Mice inoculated orally with irradiated oocysts, killed tachyzoites or excretory/secretory antigens are only partially protected against lethal *T. gondii* infection [17, 52, 53].

Research on pig vaccination against *T. gondii* incorporating mouse bioassays has not shown a strong protective response against the formation of tissue cysts in target pig tissues for inactivated/killed or multiantigen vaccines. For example, one study reported only partial protection against tissue cyst formation after pigs were intranasally immunized with *T. gondii* rhoptry proteins (200 µg/dose) and the Quil-A® adjuvant [54]. Parasite detection was observed in 5 of 11 mice (45.4%) in the vaccinated group and in 7 of 9 mice (77.8%) in the nonvaccinated group after challenge with the VEG Type III strain. Similarly, in another experiment were observed only 20% protection in pigs after two-dose subcutaneous vaccination using crude *T. gondii* rhoptry proteins—100 µg/dose—and immune-stimulating complexes (ISCOMs) as adjuvants for pig vaccination and challenge with 10 000 VEG oocysts [48]. A vaccine containing excretory–secretory antigens (ESAs)—2 mg/dose—emulsified in Freund's adjuvant administered subcutaneously showed more promising results, reporting a reduction in tissue cyst formation in pig muscles, with *T. gondii* detected in 1 of 5 vaccinated pigs (20%) compared with 5 of 5 (100%) control animals, ranging from a 38% detection frequency in unvaccinated animals to a 10.8% detection frequency in vaccinated animals after both the primary vaccination and booster doses, followed by the intraperitoneal challenge of 10^7^ tachyzoites of the GJS (Type I) strain [49]. In our study, the detection rate in mice infected with tissues from unvaccinated piglets was 43%, and it was reduced to 4–5% in mice infected with tissues from vaccinated piglets after challenge with 1000 TgShSp1 oocysts. Another study using *T. gondii* lysate antigen tested two vaccines with different protein compositions: vaccine 1, containing specific molecular weight protein fractions (20–40 kDa and 55–65 kDa) at 500 µg/dose, and vaccine 2, consisting of the complete tachyzoite lysis antigen without fractionation obtained from the RH (Type I) isolate and administered at 500 µg/dose. In this case, the results did not include a bioassay but were instead assessed using magnetic capture qPCR, which revealed the absence of detectable infection in 1 of 5 animals (20%) vaccinated with vaccine 1 and in 2 of 5 animals (40%) vaccinated with vaccine 2 after challenge with IPB-LR (Type II) tissue cysts. Notably, the booster dose was administered after the challenge [39]. Although the techniques used are not directly comparable to those used in our study, notably, the vaccine we used resulted in a more pronounced reduction in parasite detection, as determined by qPCR in the vaccinated groups, with consistently lower parasite loads than those in the control group. Furthermore, the selection of certain *T. gondii* protein fractions as antigens and the use of antigen quantities in these approaches could limit the production yield required for market implementation, which can be achieved for the production of our antigen.

To date, only a live attenuated vaccine derived from the S48 strain has displayed significant efficacy in reducing the presence of viable cysts [14]. In this study, all the mice inoculated with tissues from vaccinated pigs, which had been challenged with 1000 M4 oocysts, survived, whereas only 51.1% of the mice inoculated with tissues from non-vaccinated pigs survived. Additionally, 100% (45 of 45) of the mice inoculated with tissues from vaccinated pigs tested negative for *T. gondii* by ITS1 PCR [14]. Other studies have been conducted with live attenuated candidates with similar results [15, 16], but vaccination with a temperature-sensitive live candidate failed to prevent cyst formation in the tissues of piglets [55]. Live attenuated vaccines have shown that the cellular immune response, including IFN-γ production—a key marker of the Th1 response—is critical for controlling *T. gondii* infection [40], and as previously discussed in the context of immunogenicity in mice, a Th1-predominant immune response, characterized by high levels of IFN-γ, is considered essential for providing robust protection against *T. gondii* infection [44–46]. In this case, Quil-A® has been used as an adjuvant in vaccination because, as shown in this study and previous studies, it induces a Th1 response that is crucial for combating intracellular infections such as *T. gondii*, along with a Th2 response, thereby having the ability to modulate the immune system toward a more balanced response [37–39]. This balance is also important because if only a highly proinflammatory or Th1 response is induced, it could lead to the excessive activation of the immune system, resulting in chronic inflammation or tissue damage [56].

Regarding immunogenicity in piglets, the inactivated vaccine induced a robust humoral immune response, with significant increases in IgG, IgG1, and IgG2 levels observed beginning at 7 days after the booster immunization. These levels remained elevated compared with those in the unvaccinated groups until the time of challenge. These findings are consistent with previous studies using inactivated vaccines, where similar kinetics were observed, with vaccinated animals beginning to exhibit seroconversion between days 7 and 15 after the booster immunization and maintaining higher antibody levels than those in the control group until the time of challenge [48, 49]. However, our study produced better results than another study in which only half of the animals had seroconverted by the time of challenge [54]. Regarding cellular immune response, both vaccine doses induced a cellular immune response, as measured by IFN-γ production. IFN-γ levels were higher in animals from the vaccinated groups, with significant differences observed only in the G2–40 group at 15 days after the booster was administered (before the challenge). These findings are consistent with other studies using a subunit vaccine, in which pigs immunized with ESAs presented significantly higher IFN-γ levels than did the control pigs at 15 days after the booster immunization [49].

Notably, no statistically significant differences in efficacy were observed between the 20 µg and 40 µg vaccine doses in terms of the parasite burden. This finding suggests that as little as 20 µg of *T. gondii* proteins is sufficient to confer protection in piglets. Nonetheless, the 40 µg dose is recommended because of its superior clinical and immunological performance. Animals vaccinated with this dose presented milder clinical signs post-challenge, a more robust humoral immune response (notably higher IgG titers), and a significantly stronger IFN-γ-mediated cellular immune response during the prechallenge period. These findings indicate the increased activation of both the humoral and cellular arms of the immune system, which are critical for sustained protection against *T. gondii.*

The development of an inactivated vaccine against *T. gondii* represents a significant advancement not only in veterinary medicine but also in the broader context of the One Health paradigm by directly contributing to the reduction in zoonotic transmission [57]. Human infection is frequently linked to the consumption of undercooked or raw pork meat containing viable tissue cysts or through environmental exposure to oocysts. By reducing tissue cyst formation in infected piglets, the vaccine has the potential to mitigate one of the main foodborne pathways of transmission to humans. Furthermore, the immunological insights gained from this model could inform the future development of vaccines for high-risk human populations, including pregnant women and immunocompromised individuals. This study highlights the dual impacts of veterinary vaccination on protecting both animal and public health, reinforcing the value of integrated control measures.

In summary, this study demonstrates that the inactivated TgPigSp1 vaccine is a promising candidate to reduce *T. gondii* infection in pigs. By incorporating antigens from both tachyzoite and bradyzoite stages of a recent field isolate, this multistage vaccine approach offers a novel strategy for controlling the risk of tissue cyst formation. The vaccine elicited consistent humoral and cellular immune responses, significantly lowered parasite burden, and reduced the formation of viable tissue cysts, as confirmed by mouse bioassay and molecular detection. Importantly, the vaccine was well tolerated with no systemic adverse effects observed in vaccinated piglets.

Therefore, while live attenuated vaccines [14, 40] can achieve complete protection, their associated risks limit their practical use. Given these challenges, inactivated vaccines represent a safer alternative; however, many have shown only partial efficacy, with no total elimination of parasites in target tissues. In contrast, our inactivated vaccine reduced parasite load and limited the formation of viable tissue cysts in vaccinated animals, suggesting an improvement compared with previously reported outcomes. Although these findings are encouraging, further studies involving a greater number of animals, extended follow-up periods, and evaluation under field conditions are needed to confirm the consistency and durability of the protection. When combined with biosecurity measures, appropriate farm management and hygiene practices, this vaccine could contribute to reducing the burden of *T. gondii* in food-producing animals. While achieving *Toxoplasma*-free farms remains a long-term goal, our results support the potential role of inactivated vaccines as part of integrated control strategies to improve food safety and public health.

## Supplementary Information

**Additional file 1. LC‒MS/MS analysis of the TgShSp3 and TgPigSp1 vaccine antigens. **(A) Pie chart showing the number and percentage of *C. sabaeus* proteins and *T. gondii* proteins identified in the TGME49 and TGVEG databases. (B) Violin plot depicting the abundances of the *C. sabaeus* and *T. gondii* proteins in the vaccine antigens of TgPigSp1 and TgShSp3. The vertical continuous line in the violin plot marks the median and discontinues quartiles, **** (*p* < 0.0001; Mann‒Whitney U test). (C) Venn diagram of quantified *T. gondii* proteins in TgPigSp1 and TgShSp3 vaccine antigens showing the shared and specifically quantified proteins in the TgPigSp1 and TgShSp3 vaccine antigens. (D) Volcano plot showing that *T. gondii* protein expression significantly decreased (fold change < 0.66) or significantly increased (fold change > 1.5) in the TgPigSp1 *vs.* TgShSp3 vaccine antigens, with a p-adjusted q value < 0.05. The numbers on the volcano plot indicate that the number of differentially abundant proteins increased in TgShSp3 on the left (labeled in green) and increased in TgPigSp1 on the right (labeled in red). Note that all the differentially abundant proteins are represented according to the p-adjusted value (q value) and not filtered for the coefficient of variation (CV). (E) Abundances of identified proteins associated with the tachyzoite and bradyzoite stages of *T. gondii* identified for the TgPigSp1 and TgShSp3 vaccine antigens. * Marks differentially abundant proteins (fold change > 1.5 and < 0.7; q value > 0.05; CV<30) between the TgPigSp1 and TgShSp3 proteins.

**Additional file 2. Immunoblots showing the levels of TgShSp3 (A, C) and TgPigSp1 (B, D) vaccine antigens detected with hyperimmune mouse sera against *T. gondii* (A, B) and with anti-BAG1 protein (C, D). **The number over each lane in the gel images is the number of passages of the batch of vaccine antigens produced by TgShSp3 (A) and (C) and TgPigSp1 (B) and (D). The white arrows on the right of the image gels indicate the relative molecular weights of the Precision Plus Protein Standards Kaleidoscope^TM^ marker in kDa. The black and blue arrows on the left of the image gels in (A) and (B) indicate immunoreactive bands recognized by Quantity software and their relative molecular weights in kDa. Black and blue indicate common and specific immunoreactive bands, respectively, for the TgShSp3 (A) and TgPigSp1 (B) vaccine antigens. * The gel images in (C) and (D) indicate the BAG-1 immunoreactive band with a relative molecular weight of 28 kDa.

**Additional file 3.** **Scoring of systemic effects and local reactions in the mouse following vaccination and booster.**

**Additional file 4. Levels of anti-*T. gondii* IgG1 (A) and IgG2a (B) and the ratio of IgG1/IgG2a (C) throughout the piglet trial.** Anti-*T. gondii* IgG1 (A) and IgG2a (B) levels are presented as the relative index percent (RIPC) obtained from an in-house ELISA. The vertical bars indicate the standard deviations. Asterisks above the data points denote significant differences between the immunized groups and the G3–PBS group: *p < 0.05, **p < 0.01, ***p< 0.001, and ****p < 0.0001; two-way ANOVA. (C) The graph shows the IgG1/IgG2a ratio during the trial for each group. The horizontal line represents the median; boxes indicate the interquartile range; and whiskers represent the minimum and maximum values. On the X-axis, the black arrow indicates the time point at which antibody levels increased in vaccinated animals, whereas the red arrow marks the seroconversion of the G3 group of animals after challenge.

**Additional file 5. Individual IgG and IFN-γ responses during the vaccination and booster periods in animals from the vaccinated groups. **The graphs represent (A, G1—20 and B, G2—40) IgG antibody levels reported as the relative percentage index (RIPC) obtained from an in-house ELISA per animal and (C, G1—20 and D, G2—40) IFN-γ levels (pg/mL) for each animal. The line represents the median value, and animals marked with a star indicate those that tested positive for *T. gondii* in at least one tissue by the mouse bioassay. The orange stars in graphs A and C represent piglet number 5.

**Additional file 6. Vaccine antigen characterization.**

**Additional file 7.** **Scoring of systemic effects and local reactions in the piglet following vaccination and booster.**

## Data Availability

All data generated or analyzed during this study are included in this published article and its supplementary information files. The dataset generated during the current study is available in the Zenodo repository at: 10.5281/zenodo.15672730.
