# Supplementary figures and images for: An inactivated vaccine against acquired Toxoplasma gondii infection in pigs as a tool to minimize the zoonotic transmission risk
Source: Vet Res. 2025 Oct 30;56:206. doi: 10.1186/s13567-025-01645-2 (PMC12577071; doi:10.1186/s13567-025-01645-2)

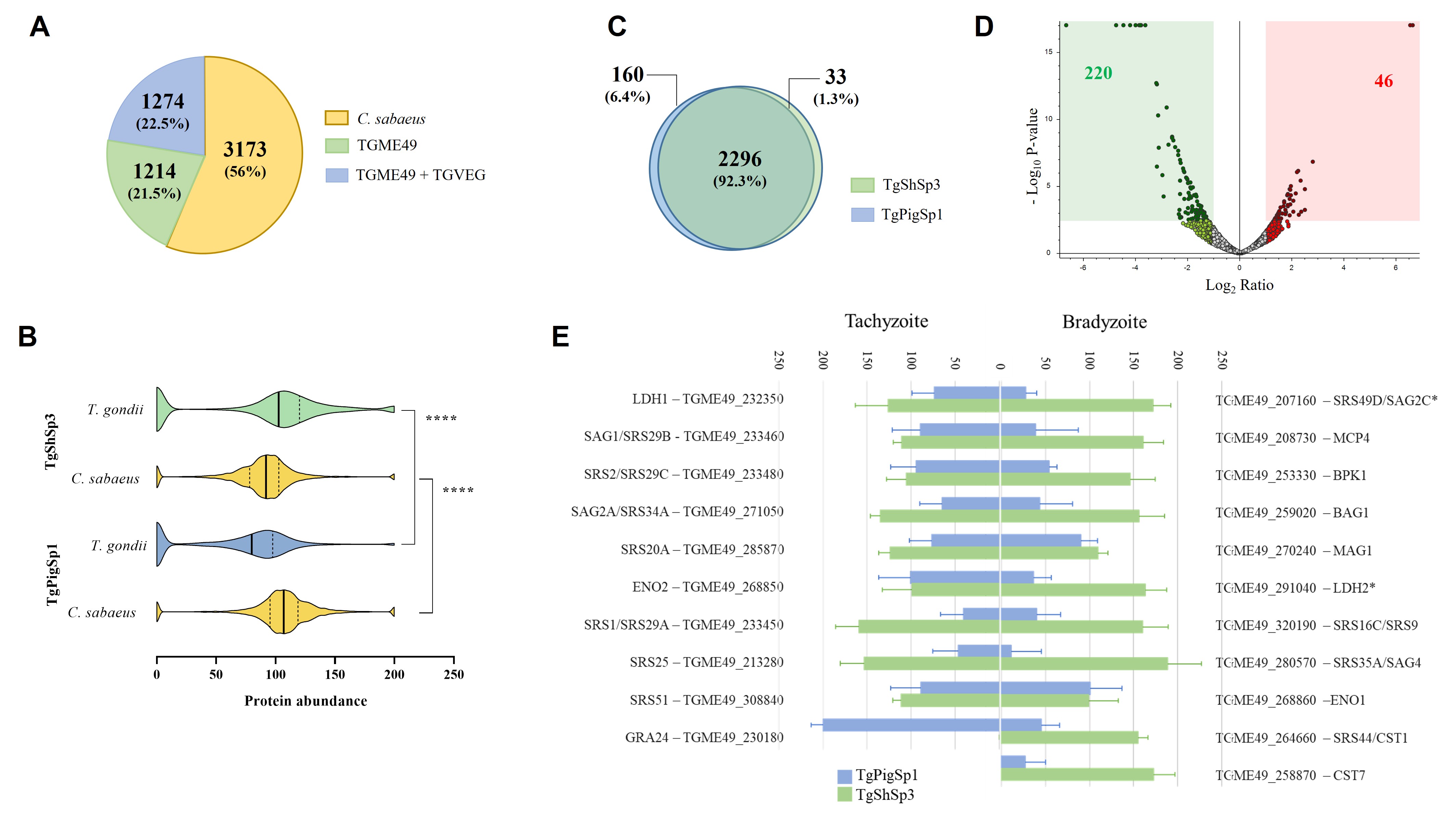

Supplement: Supplementary file 1 — Additional file 1. LC‒MS/MS analysis of the TgShSp3 and TgPigSp1 vaccine antigens. (A) Pie chart showing the number and percentage of C. sabaeus proteins and T. gondii proteins identified in the TGME49 and TGVEG databases. (B) Violin plot depicting the abundances of the C. sabaeus and T. gondii proteins in the vaccine antigens of TgPigSp1 and TgShSp3. The vertical continuous line in the violin plot marks the median and discontinues quartiles, **** (p < 0.0001; Mann‒Whitney U test). (C) Venn diagram of quantified T. gondii proteins in TgPigSp1 and TgShSp3 vaccine antigens showing the shared and specifically quantified proteins in the TgPigSp1 and TgShSp3 vaccine antigens. (D) Volcano plot showing that T. gondii protein expression significantly decreased (fold change < 0.66) or significantly increased (fold change > 1.5) in the TgPigSp1 vs. TgShSp3 vaccine antigens, with a p-adjusted q value < 0.05. The numbers on the volcano plot indicate that the number of differentially abundant proteins increased in TgShSp3 on the left (labeled in green) and increased in TgPigSp1 on the right (labeled in red). Note that all the differentially abundant proteins are represented according to the p-adjusted value (q value) and not filtered for the coefficient of variation (CV). (E) Abundances of identified proteins associated with the tachyzoite and bradyzoite stages of T. gondii identified for the TgPigSp1 and TgShSp3 vaccine antigens. * Marks differentially abundant proteins (fold change > 1.5 and < 0.7; q value > 0.05; CV<30) between the TgPigSp1 and TgShSp3 proteins. [file 13567_2025_1645_MOESM1_ESM.jpg]

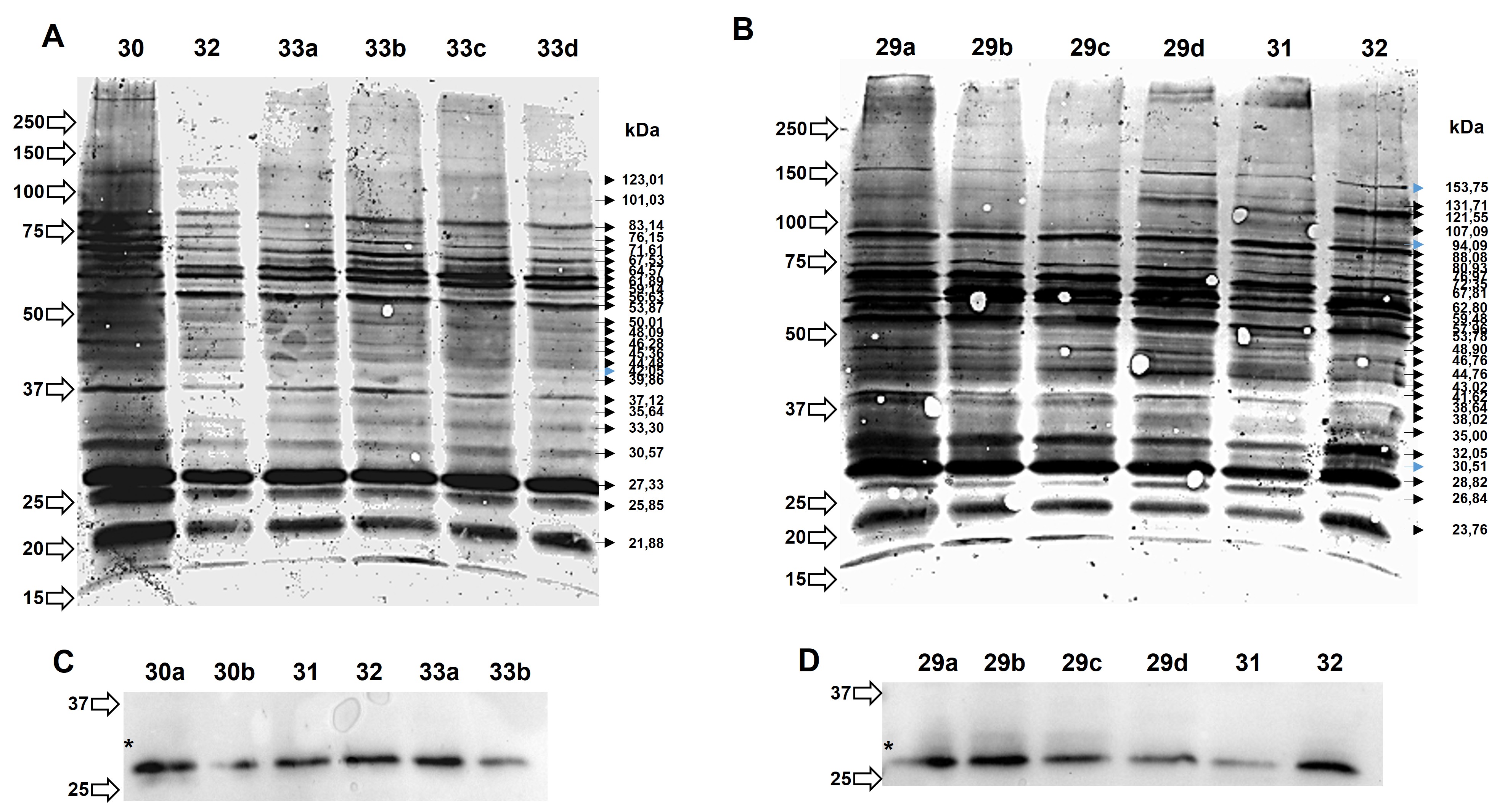

Supplement: Supplementary file 2 — Additional file 2. Immunoblots showing the levels of TgShSp3 (A, C) and TgPigSp1 (B, D) vaccine antigens detected with hyperimmune mouse sera against T. gondii (A, B) and with anti-BAG1 protein (C, D). The number over each lane in the gel images is the number of passages of the batch of vaccine antigens produced by TgShSp3 (A) and (C) and TgPigSp1 (B) and (D). The white arrows on the right of the image gels indicate the relative molecular weights of the Precision Plus Protein Standards KaleidoscopeTM marker in kDa. The black and blue arrows on the left of the image gels in (A) and (B) indicate immunoreactive bands recognized by Quantity software and their relative molecular weights in kDa. Black and blue indicate common and specific immunoreactive bands, respectively, for the TgShSp3 (A) and TgPigSp1 (B) vaccine antigens. * The gel images in (C) and (D) indicate the BAG-1 immunoreactive band with a relative molecular weight of 28 kDa. [file 13567_2025_1645_MOESM2_ESM.jpg]

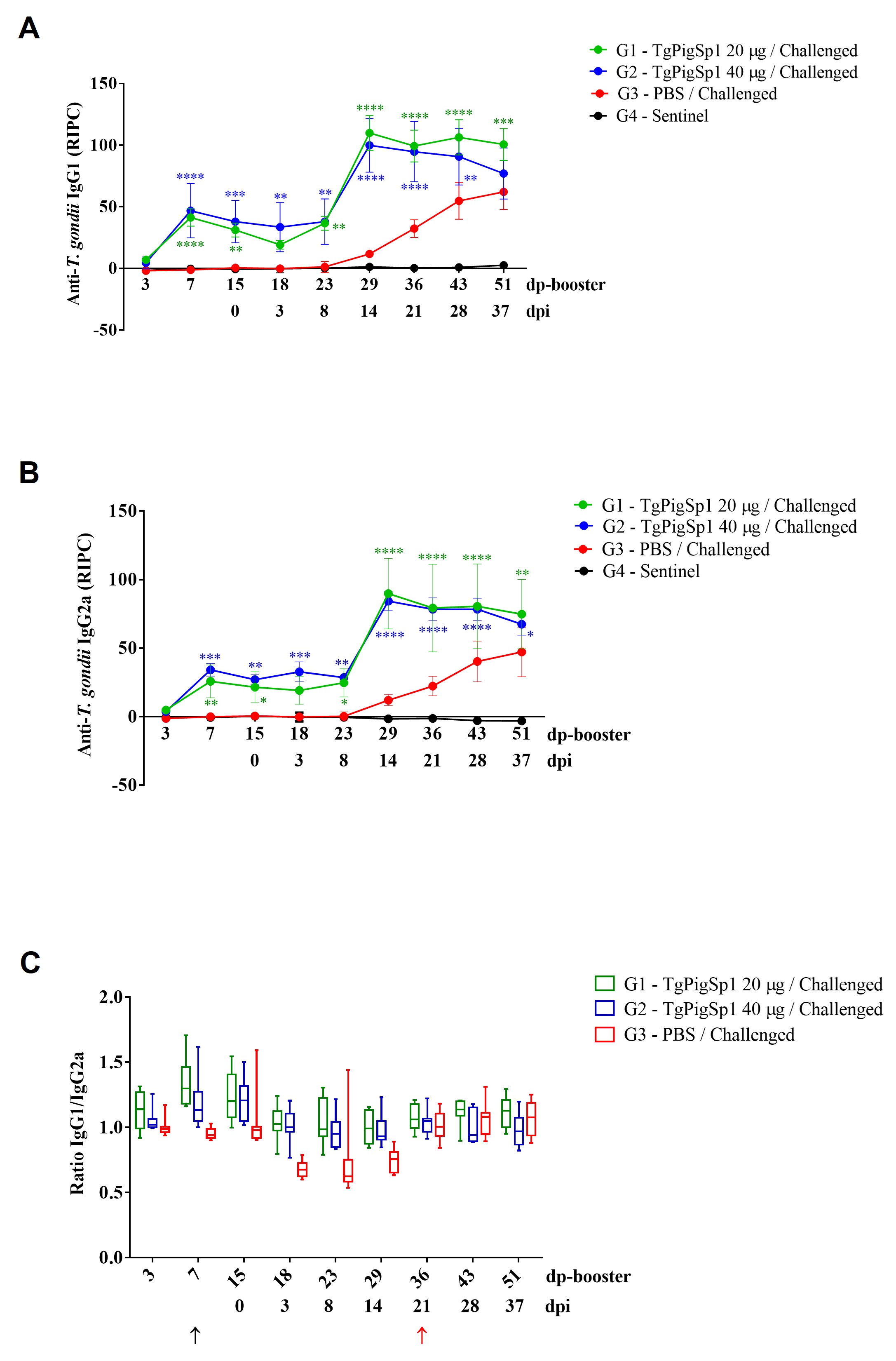

Supplement: Supplementary file 4 — Additional file 4. Levels of anti-T. gondii IgG1 (A) and IgG2a (B) and the ratio of IgG1/IgG2a (C) throughout the piglet trial. Anti-T. gondii IgG1 (A) and IgG2a (B) levels are presented as the relative index percent (RIPC) obtained from an in-house ELISA. The vertical bars indicate the standard deviations. Asterisks above the data points denote significant differences between the immunized groups and the G3–PBS group: *p < 0.05, **p < 0.01, ***p< 0.001, and ****p < 0.0001; two-way ANOVA. (C) The graph shows the IgG1/IgG2a ratio during the trial for each group. The horizontal line represents the median; boxes indicate the interquartile range; and whiskers represent the minimum and maximum values. On the X-axis, the black arrow indicates the time point at which antibody levels increased in vaccinated animals, whereas the red arrow marks the seroconversion of the G3 group of animals after challenge. [file 13567_2025_1645_MOESM4_ESM.jpg]

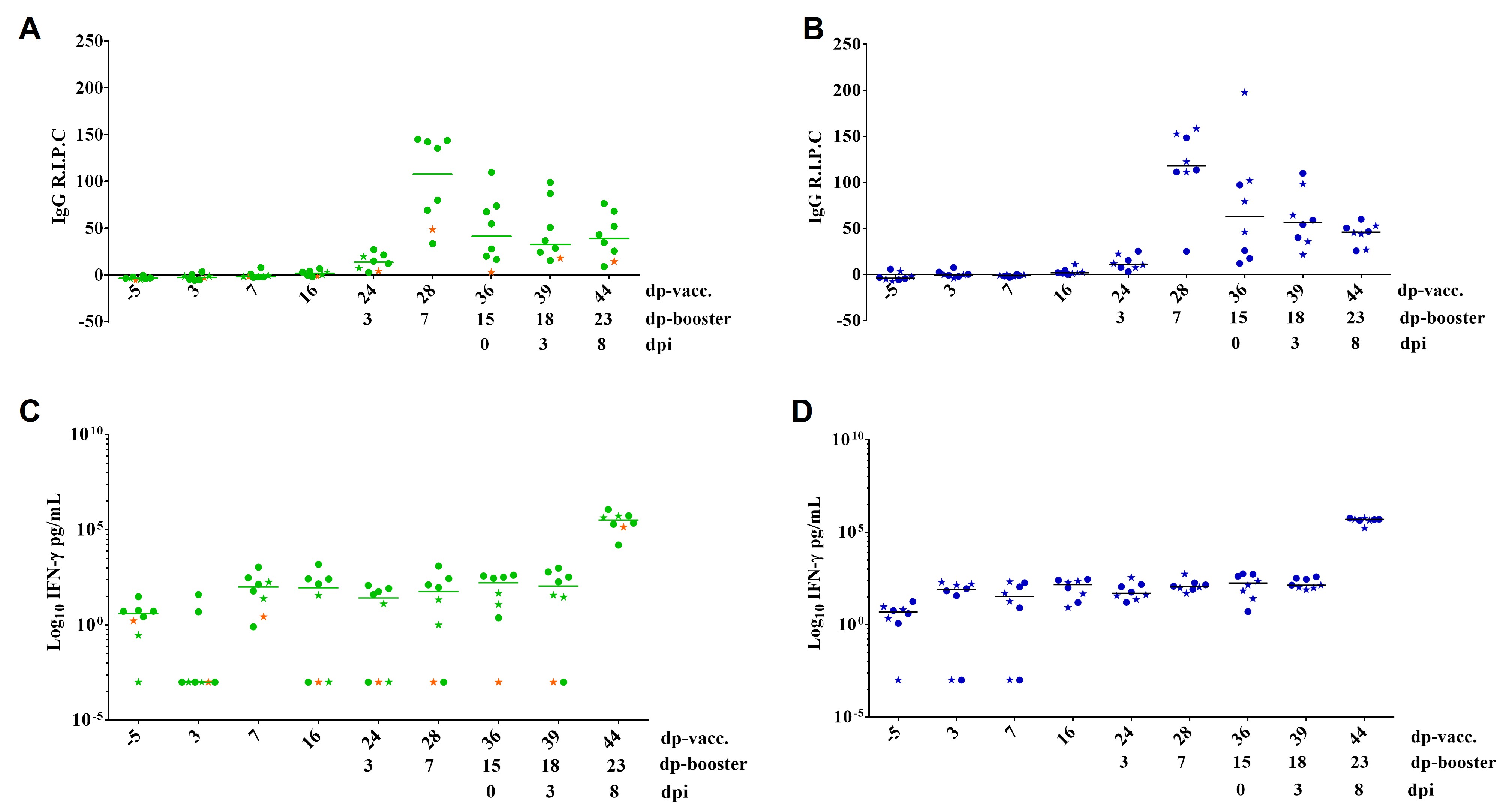

Supplement: Supplementary file 5 — Additional file 5. Individual IgG and IFN-γ responses during the vaccination and booster periods in animals from the vaccinated groups. The graphs represent (A, G1—20 and B, G2—40) IgG antibody levels reported as the relative percentage index (RIPC) obtained from an in-house ELISA per animal and (C, G1—20 and D, G2—40) IFN-γ levels (pg/mL) for each animal. The line represents the median value, and animals marked with a star indicate those that tested positive for T. gondii in at least one tissue by the mouse bioassay. The orange stars in graphs A and C represent piglet number 5. [file 13567_2025_1645_MOESM5_ESM.jpg]
