# Supplementary material for: An inactivated vaccine against acquired Toxoplasma gondii infection in pigs as a tool to minimize the zoonotic transmission risk
Source: Vet Res. 2025 Oct 30;56:206. doi: 10.1186/s13567-025-01645-2 (PMC12577071; doi:10.1186/s13567-025-01645-2)
Supplement: Supplementary file 3 — Additional file 3. Scoring of systemic effects and local reactions in the mouse following vaccination and booster. [file 13567_2025_1645_MOESM3_ESM.pdf]

Additional file 3: Scoring of systemic effects and local reactions in the mouse following vaccination and booster.

Systemic effects	Graduation	Nodules/lesions	Graduation
No alterations	0	Absent	0
Ruffled coat	1	Small size (< 2.5 mm in diameter)	1
Ruffled coat + decreased activity < 24 h	2	Medium size (< 5 mm)	2
Ruffled coat + decreased activity > 24h < 48 h	3	Large size (< 10 mm)	3
Ruffled coat + decreased activity > 48 h	4	Very large size (> 10 mm)	4
