# Supplementary material for: An inactivated vaccine against acquired Toxoplasma gondii infection in pigs as a tool to minimize the zoonotic transmission risk
Source: Vet Res. 2025 Oct 30;56:206. doi: 10.1186/s13567-025-01645-2 (PMC12577071; doi:10.1186/s13567-025-01645-2)
Supplement: Supplementary file 6 — Additional file 6. Vaccine antigen characterization. [file 13567_2025_1645_MOESM6_ESM.pdf]

Additional file 6. Vaccine antigen characterization.

An inactivated vaccine against porcine toxoplasmosis to reduce the risk of human infection through meat consumption.

Largo de la Torre, A[1]; Sánchez-Sánchez, R[2]; Diezma-Díaz, C[1]; Ferre, I[2], Regidor-Cerrillo, J[1*†]., Ortega-Mora L.M[2*†.]

Vaccine protein composition by liquid chromatography-tandem mass spectrometry (LC-MS/MS) analysis.

In-Gel Digestion

Vaccine antigen samples were run in SDS-PAGE gel. Samples in Laemmli sample buffer were loaded into a 1.5 mm thick SDS-PAGE gel with a 4% stacking gel casted over a 10% resolving gel. The run was stopped as soon as the front entered 3 mm into the resolving gel so that the whole proteome became concentrated in the stacking/resolving gel interface. Band were stained with Coomassie and cut for protein digestion in gel with trypsin solution. Briefly, proteins were reduced with 10 mM DTT at 56 °C for 30 minutes and alkylated in the dark for 20 minutes with 55 mM iodoacetamide. Then, samples were digested at 37 °C overnight with a 1/50 (w/w) of Recombinant Sequencing Grade Trypsin (Roche Molecular Biochemicals, Mannheim, Germany) in 25 mM ammonium bicarbonate pH 8.5. The resulting peptides were eluted with 80% acetonitrile (ACN)/0.1% trifluoroacetic acid (TFA), dried in a vacuum centrifuge, and finally resuspended in 25 µL of 0.1% formic acid (FA)/2% ACN and quantified by Qubit fluorometer (Thermo-Scientific).

MS Analysis

Peptides were analyzed by nano LC-MS/MS using a nano Easy-nLC 1000 couple to a high-resolution mass spectrometer Q-exactive HF (Thermo-Scientific). The digested peptide mixtures were concentrated (on-line) by RP chromatography using an Acclaim PepMap 1000 precolumn (20 mm x 75 µm ID, C18, 3 µm silica particles, 100 Å pore size - Thermo Scientific), and then, peptides were separated using a C18 Picofrit analytical column (500 mm x 75 µm ID, Easy Spray Column, PepMap RSLC C18n, 2 µm particles, 100 Å pore size - Thermo Scientific) and eluted using a 150 minutes gradient from 5% solvent B to 35% solvent B in A, 10 minutes gradient from 35% to 45% solvent B in solvent A, 1 minute gradient from 45% to 95% solvent B in solvent A and 25 minutes gradient of 95% solvent B in solvent A (solvent A contained 0.1% AF, 2% ACN in water; solvent B contained 0.1% AF in ACN), operating at 250 nL/min. Peptides eluting from the column were electrosprayed directly into the mass spectrometer (on-line) from the analytical column with an ion transfer tube at temperature of 180 °C. The peptides were detected with a resolution of 60,000 in Full scan MS mode on a m/z mass range of 300-1,800 Da. MS/MS data were acquired in data-dependent acquisition mode of the MS and in each microscan, depending on of its intensity (threshold: $2x10^3$) up to 15 precursors with a charge from 2+ to 4+ were selected with dynamic exclusion of 10 seconds, using

an isolation with a window width of +/- 2 units of m/z and in a maximum time of 120 ms, for its fragmentation by high collision dissociation with an energy of normalized collision of 20%. MS/MS spectra were acquired in positive mode.

Protein/Peptide Identification and Quantification

Peptide and protein identification from mass spectra raw data was carried out through Proteome Discoverer 2.4 (Thermo Scientific) using licensed version of search engine MASCOT 2.6.1 (Matrix Science, London, UK). Tandem MS/MS data were searched against database with predicted sequences of *T. gondii* downloaded August 2021 from TOXODB (https://toxodb.org/toxo/app/downloads/release-51/TgondiiME49/fasta/data/; Toxoplasma gondii 51: ME49; 8322 sequences), and database of *Chlorocebus sabaeus* from Uniprot (www.uniprot.org; 19229 sequences) and the data base Contaminants (247 sequencies) from Max Planck Institute of Biochemistry (https://www.biochem.mpg.de/mass_spectrometry). Search parameters included a maximum of two missed tryptic cleavages after Arg and Lys allowed, carbamidomethylation of cysteines as a fixed modification, and optional oxidation of methionine, loss of acetylation or Met + acetylation of the N-terminal end of the protein as variable modifications. Precursor and fragment mass tolerance were set to 10 ppm and 0.02 Da, respectively. Search against decoy database (Mascot integrated decoy approach) was used to FDR calculation. MASCOT percolator filter was applied to MASCOT results (Matrix Science). The acceptances criteria for proteins identification were a false discovery rate (FDR) < 1% and, at least, one unique peptide identified with high confidence (Confidence Interval CI > 99%).

To determine the abundances of the identified peptides and proteins, recalibration of masses was carried out by means of a quick search with Sequest HT against the corresponding *T. gondii* database and, based on the identifications, taking into account the peptides that are present in at least 50% of the replicates, an alignment of the chromatograms of all the samples with a tolerance of up to 10 minutes. Finally, the total amount of protein between the different samples is normalized using the total abundance of all the peptides. For comparisons of protein abundances between TgShSp3 and TgPigSp1 vaccine antigen, only unique peptides without variables modifications are used to protein ratios calculation using a non-nested/pairwise design. The protein ratios were calculated as the median of all combinations of ratios from biological replicates for the identified unique peptides. The protein abundance ratio is then calculated as the median of the peptide group ratios. Proteins differentially abundant between TgShSp3 and TgPigSp1 vaccine antigen were considered those with a p-adjusted (q value) < 0.05; and a fold change ≥ 2 for more abundant and ≤0.5 for less abundant ($\log_2$ ratio ≥ 1 and $\log_2$ ratio ≤ - 1, respectively) with a coefficient of variation ≤ 30.

Immunobloting profiles of vaccine antigen.

Immunoblotting profile of vaccine antigen was studied in nitrocellulose membranes after protein transference of 12 % SDS-PAGE gels run with 10 µg of protein of vaccine antigen of the different batches of production in a Mini-PROTEAN Tetra Cell system (Biorad). For SDS—PAGE gels the amount of the vaccine antigen from each batch production was mixed with the required amount of protein lysis buffer 2× (sodium dodecyl sulfate –SDS-

4%, glycerol 10%, 60 mM of Tris-HCl (pH 6.8), 100 mM of dithiothreitol, and 0.048% of bromophenol blue), boiled during 5 min and run in SDS-PAGE in parallel with the Precision Plus Protein Standards Kaleidoscope TM marker (Bio-Rad, Hercules, California, USA) to determine the relative molecular weight of the immunoreactive bands. The different samples were resolved at 100 V (constant) during 2 h in a bis/acrylamide stacking gel at 4% (pH 6.8), followed by an acrylamide/bisacrylamide separating gel at 12%, in the presence of Tris-Glycine-SDS electrophoresis buffer. Then, proteins were transferred in the same system to membranes at 0.3A (constant) during 1.5 h in a Tris-Glycine-Methanol buffer. After blockade with tris-buffered saline with 0.05 % Tween® 20 detergent (TBST, Sigma-Aldrich®) plus 5 % skimmed milk, the membranes were incubated for one hour with a hyperimmune mouse antiserum directed against *T. gondii* (1:50). Mouse sera was obtained by a mixture of equal volume of the serum of seropositive animals infected with 100 oocysts of the TgShSp1 isolate of Type II and 500 oocysts of the TgShSp24 isolate of Type III, which had been obtained from previous mouse experimental infections (Largo de la Torre et al., 2025). Then, after three washes with TBST, membranes were incubated with an anti-mouse IgG2a conjugated HRP at dilution 1:2,000 (1070-05, SouthernBiotech), and after three washes with TBST and two with TBS (without Tween® 20 detergent) membranes were revealed by chemiluminescence using the ECL substrate (Thermo-Fisher). Antigen profile analyses were carried out comparing the relative molecular weight of stained bands determined with the software Quantity One (Bio-Rad) and the Precision Plus Protein Standards KaleidoscopeTM marker (Bio-Rad) as reference. Presence of specific bradyzoite protein TgBAG1 in vaccine antigen was also checked by immunoblotting in membranes incubated with a monoclonal mouse serum against TgBAG1 at 1:100 dilution (Genscript, the Netherlands).
