# Supplementary material for: An inactivated vaccine against acquired Toxoplasma gondii infection in pigs as a tool to minimize the zoonotic transmission risk
Source: Vet Res. 2025 Oct 30;56:206. doi: 10.1186/s13567-025-01645-2 (PMC12577071; doi:10.1186/s13567-025-01645-2)
Supplement: Supplementary file 7 — Additional file 7. Scoring of systemic effects and local reactions in the piglet following vaccination and booster. [file 13567_2025_1645_MOESM7_ESM.pdf]

Additional file 7. Scoring of systemic effects and local reactions in the piglet following vaccination and booster.

Systemic effects	Graduation	Nodules/lesions	Graduation
No alterations	0	Absent	0
Fever	1	Edema / Inflammation	1
Fever + decrease appetite	2	Inflammation + redness	2
Fever + decrease appetite + decreased activity	3	Pain	3
